# In Silico Design and Evaluation of a Multi-Epitope Vaccine Candidate Against *Staphylococcus haemolyticus*

**DOI:** 10.3390/microorganisms14102152

**Published:** 2026-09-24

**Authors:** Ahmad Almatroudi

**Affiliations:** Department of Medical Laboratories, College of Applied Medical Sciences, Qassim University, Buraydah 51452, Saudi Arabia; aamtrody@qu.edu.sa

**Keywords:** *Staphylococcus haemolyticus*, multi-epitope vaccine, antimicrobial resistance, reverse vaccinology, immunoinformatics

## Abstract

*S. haemolyticus* is an emerging multidrug-resistant pathogen that frequently causes nosocomial infections, yet no licensed vaccine is currently available for its prevention. In this study, conserved surface-associated proteins were screened to identify B-cell, cytotoxic T-lymphocyte, and helper T-lymphocyte epitopes for the design of a multi-epitope vaccine candidate. The selected epitopes were assembled into a 254-amino acid construct containing human β-defensin-3, the PADRE sequence, and selected linkers. The resulting construct was predicted to be antigenic, non-allergenic, and non-toxic, with favorable physicochemical properties, structural stability, and solubility. The selected CTL and HTL epitopes provided an estimated global population coverage of 97.5%, indicating broad predicted coverage across different populations. Structural analysis supported the quality of the refined three-dimensional model. Molecular docking predicted favorable interactions between the vaccine construct and TLR2 and TLR4, while 200 ns molecular dynamics simulations indicated that receptor–vaccine interactions were maintained during the simulation period. Codon optimization and in silico cloning further supported the feasibility of recombinant expression in *Escherichia coli*. Overall, the computational analyses support the proposed multi-epitope construct as a potential vaccine candidate against *S. haemolyticus* and provide a basis for its further experimental evaluation.

## 1. Introduction

Nosocomial infections caused by coagulase-negative staphylococci (CoNS) are increasingly recognized as an important clinical concern [1]. CoNS, a group of bacteria that were once regarded as harmless commensals, are now widely recognized as clinically relevant opportunistic pathogens [2]. These organisms predominantly affect patients with prolonged hospital stays, implanted medical devices, or weakened immune status, where they contribute to a broad spectrum of difficult-to-manage infections [1]. *Staphylococcus haemolyticus*, a member of the normal human skin microbiota, has increasingly been reported in bloodstream infections, catheter-associated infections, prosthetic device infections, surgical site infections, urinary tract infections, and neonatal infections [3,4]. The ability of *S. haemolyticus* to persist in clinical settings and cause infections at different anatomical sites highlights the need for effective preventive strategies against this pathogen [5,6].

One of the defining virulence traits of *S. haemolyticus* is its ability to establish biofilms on both living tissues and inert materials [7]. These highly organized microbial structures are composed of densely packed bacterial cells enclosed within an extracellular polymeric matrix synthesized by the bacteria themselves, which promotes survival and persistence under adverse environmental conditions [6]. The recurrent nature of these infections and the difficulty of eliminating biofilm-associated bacteria emphasize the need for preventive approaches that complement conventional antimicrobial therapy [7]. Host defense against bacterial infection involves coordinated innate and adaptive immune responses, including recognition of microbial components, activation of antigen-presenting cells, production of inflammatory mediators, and generation of antigen-specific B-cell and T-cell responses. Effective stimulation of these immune mechanisms is therefore an important consideration in the development of preventive vaccines [8,9].

The clinical management of *S. haemolyticus* infections is further complicated by the increasing occurrence of multidrug-resistant isolates [10]. Clinical investigations have demonstrated that *S. haemolyticus* isolates can exhibit resistance to multiple antimicrobial agents, including oxacillin, imipenem, aminoglycosides, and erythromycin [11]. Although antimicrobial therapy remains an important treatment option, antimicrobial resistance and biofilm formation can complicate the treatment of infections and contribute to their persistence [12]. Thus, antimicrobial resistance represents an additional challenge in the management of *S. haemolyticus* infections and shows the importance of preventive approaches. Although the medical importance of *S. haemolyticus* continues to increase, no licensed vaccine is currently available specifically for this pathogen [13]. This lack of an available vaccine highlights the value of investigating vaccination as an additional preventive measure to complement existing approaches for controlling *S. haemolyticus* infections.

Advances in genome-based vaccine discovery have provided new opportunities to identify vaccine candidates using computational methods [14]. Reverse vaccinology enables the analysis of pathogen genomes and proteomes to identify potential vaccine antigens, whereas immunoinformatics can be used to identify and evaluate B-cell and T-cell epitopes [15,16]. Multi-epitope vaccine design combines selected immunogenic epitopes into a single construct, allowing different B-cell and T-cell epitopes to be incorporated into one vaccine candidate [17]. In addition, computational analyses such as structural modeling, molecular docking, molecular dynamics simulations, and other immunoinformatics approaches can provide preliminary information about the predicted structural and immunological properties of vaccine constructs before laboratory evaluation [18,19].

This study used reverse vaccinology and immunoinformatics to design a vaccine candidate against *S. haemolyticus*. Conserved surface-associated proteins were screened for suitable epitopes, which were then assembled into a chimeric vaccine construct. The construct was evaluated for antigenicity, allergenicity, toxicity, physicochemical properties, structural characteristics, receptor interactions, conformational stability, codon adaptation, and in silico cloning. These computational analyses provide preliminary support for further experimental evaluation of the proposed vaccine candidate against this clinically significant pathogen.

## 2. Materials and Methods

The complete methodological workflow adopted for the design and evaluation of the multi-epitope vaccine is presented in Figure 1. The figure outlines the computational steps, from target protein selection and epitope identification to structural validation and in silico cloning.

### 2.1. Selection and Screening of Surface Proteins

The completeproteome of *S. haemolyticus* was obtained from the National Center for Biotechnology Information (NCBI) Protein database (https://www.ncbi.nlm.nih.gov/, accessed on 20 July 2026). Proteins annotated as surface-exposed were extracted and used as the initial dataset for vaccine target identification. Antigenicity was assessed using VaxiJen v2.1 (https://www.ddg-pharmfac.net/vaxijen/, accessed on 22 July 2026) [20], using the bacterial prediction model with an antigenicity cutoff of 0.4. Proteins with antigenicity scores above the predefined cutoff were retained for further analysis. The allergenic potential of the retained proteins was evaluated using AllerTOP v2.1 (http://www.ddg-pharmfac.net/AllerTOP/, accessed on 25 July 2026) [21], and proteins predicted to be allergenic were excluded. The remaining candidate proteins were further screened for characteristics relevant to vaccine target selection. Subcellular localization was predicted using CELLO v2.5 (http://cello.life.nctu.edu.tw/, accessed on 23 July 2026) [22] to assess their predicted surface or extracellular localization. SignalP 6.0 (https://services.healthtech.dtu.dk/services/SignalP-6.0/, accessed on 23 July 2026) was used to identify N-terminal signal peptides associated with the classical secretion pathway [23]. TMHMM v2.0 (https://services.healthtech.dtu.dk/services/TMHMM-2.0/, accessed on 23 July 2026) was used to predict transmembrane helices and determine the membrane topology of the candidate proteins [24]. Proteins containing 0–1 predicted transmembrane helix were retained for subsequent analyses. To assess potential sequence similarity with human proteins, each candidate protein was compared with the *Homo sapiens* proteome (Taxonomy ID: 9606) using NCBI BLASTp (https://blast.ncbi.nlm.nih.gov/Blast.cgi?PAGE=Proteins, accessed on 25 July 2026). Candidates without significant sequence homology to human proteins were retained for subsequent analyses. The final selected surface-associated proteins were peptide ABC transporter substrate-binding protein (NCBI accession: QCY38924.1), polysaccharide deacetylase (NCBI accession: QCY37413.1), LPXTG cell wall anchor domain-containing protein (NCBI accession: QCY37432.1), and YSIRK-type signal peptide-containing protein (NCBI accession: QCY37300.1). The sequence conservation of the selected proteins was subsequently assessed to identify conserved vaccine targets. Each protein was searched against the NCBI Protein database using BLASTp, and homologous protein sequences with more than 90% sequence identity and more than 90% query coverage were retrieved. The resulting sequences were aligned, and sequence variability was assessed by generating Shannon entropy plots using BioEdit v7.7.1. Proteins showing low sequence variability among the retrieved homologous sequences were selected for subsequent epitope prediction and vaccine construction.

### 2.2. Identification of Epitopes

Selected surface-associated proteins were used for epitope identification. Linear B-cell epitopes were predicted using BepiPred-3.0 through the IEDB Next-Generation Tools platform (https://nextgen-tools.iedb.org/, accessed on 27 July 2026) [25]. The prediction was performed using the majority-vote scoring option, a window size of 9, and ESM-2-based sequence encoding, with the sequence-length feature included in the ESM encoding. BepiPred-3.0 uses ESM-2 protein language model embeddings and provides prediction scores for linear B-cell epitopes. CTL epitopes were predicted using the NetMHCpan 4.1 EL algorithm through the IEDB Next-Generation Tools platform (https://nextgen-tools.iedb.org/pipeline?tool=tc1, accessed on 27 July 2026) [26]. The IEDB-recommended panel of 27 HLA class I alleles was selected for the prediction. The 27-allele panel represents frequently occurring HLA class I alleles in the global population. Peptides with a percentile rank below 1 were retained for subsequent analysis. Candidate HTL epitopes were predicted with the NetMHCIIpan EL v4.1 algorithm available through the IEDB Next-Generation Tools platform (https://nextgen-tools.iedb.org/pipeline?tool=tc2, accessed on 27 July 2026) [27]. The default HLA class II reference allele panel was used, and peptides with percentile ranks below 10 were retained for subsequent analysis. The selected B-cell, CTL, and HTL epitopes were subsequently evaluated for antigenicity, allergenicity, and toxicity. Antigenicity was assessed using VaxiJen v2.1 with the bacterial prediction model and an antigenicity threshold of 0.4. Only epitopes exceeding this threshold were retained. The allergenicity of the epitopes was examined with AllerTOP v2.1, while toxicity was determined using ToxinPred v1.0 (https://webs.iiitd.edu.in/raghava/toxinpred/, accessed on 30 July 2026). Only peptides simultaneously identified as antigenic, non-allergenic, and non-toxic were selected for inclusion in the multi-epitope vaccine construct. Sequence homology analysis was performed for the selected vaccine epitopes against *Homo sapiens* reference proteins (Taxonomy ID: 9606) using the NCBI BLASTp tool. The analysis was conducted against the RefSeq protein database to identify potential sequence similarities between the vaccine epitopes and human proteins. BLASTp hits were evaluated based on E-value, sequence identity, and alignment length. An E-value ≤ 0.001 was considered indicative of significant sequence homology. The resulting alignments were examined to determine the extent of similarity between each selected epitope and the corresponding human protein sequences. Only epitopes that satisfied the predefined vaccine-design criteria and did not show relevant sequence similarity with human proteins were retained for incorporation into the multi-epitope vaccine construct.

### 2.3. Epitope Conservancy Analysis

Epitope conservancy was assessed using the IEDB Epitope Conservancy Analysis tool (https://tools.iedb.org/conservancy/, accessed on 2 August 2026) [28] for the predicted B-cell, CTL, and HTL epitopes. The homologous protein sequences obtained through BLASTp analysis for the protein conservancy assessment were also used for epitope conservancy analysis. These sequences corresponded to the four selected surface proteins and included homologs showing more than 90% sequence identity and more than 90% query coverage. The resulting homologous protein sequences were used to assess the conservation of the predicted B-cell, CTL, and HTL epitopes.

### 2.4. Assessment of Population Coverage

The selected CTL and HTL epitopes were evaluated with the IEDB Population Coverage tool (https://tools.iedb.org/population/, accessed on 4 August 2026) to estimate their representation across global human populations [29]. HLA allele combinations obtained during epitope prediction were submitted to the server, while the analysis was performed using the default settings. The population coverage was calculated based on the selected HLA alleles and their reported frequencies in different geographical regions and ethnic groups. 

### 2.5. Vaccine Construct Design

The multi-epitope vaccine construct was assembled by joining the selected epitopes into a single amino acid chain [30]. Human β-defensin-3 was incorporated at the N-terminus of the vaccine construct as an immunostimulatory adjuvant and linked to the epitope region through an EAAAK linker. EAAAK was selected to provide structural separation between the adjuvant and epitope regions [31]. AAY linkers were inserted between adjacent CTL epitopes to facilitate proteasomal processing and subsequent presentation of the epitopes through MHC-I molecules, thereby supporting efficient processing of individual CTL epitopes [32]. Individual HTL epitopes were connected using GPGPG linkers to minimize the generation of junctional epitopes and support appropriate processing of the individual HTL epitopes, while maintaining separation between neighboring HTL regions [33]. KK linkers were introduced between adjacent B-cell epitopes to maintain separation between consecutive epitopes and facilitate their processing as individual antigenic regions, thereby reducing potential interference between neighboring B-cell epitopes [34]. The PADRE peptide was incorporated after the B-cell epitopes near the C-terminus of the vaccine construct through an EAAAK linker to provide structural separation between PADRE and the adjacent epitope region [35]. At the C-terminus, a hexahistidine (6×His) tag was incorporated to enable purification of the recombinant protein following heterologous expression. After construction of the complete sequence, computational assessment of antigenicity, allergenicity, and toxicity was performed using VaxiJen v2.1, AllerTOP v2.1, and the NeuroSnap ToxinPred Peptide Toxicity Prediction server (https://neurosnap.ai/service/ToxinPred%20Peptide%20Toxicity%20Prediction, accessed on 7 August 2026), respectively. These analyses were performed using the default parameters specified by the corresponding prediction servers [36].

### 2.6. Physicochemical Property Analysis

Physicochemical properties of the designed vaccine construct were analyzed using the ExPASy ProtParam server (https://web.expasy.org/protparam/, accessed on 7 August 2026) [37]. The analysis provided physicochemical parameters describing the molecular and compositional properties of the construct. These predicted parameters were used to assess the physicochemical characteristics of the designed construct before experimental evaluation. In addition, the probability of obtaining a soluble recombinant protein in *Escherichia coli* was predicted using SoluProt v1.0 (https://loschmidt.chemi.muni.cz/soluprot/, accessed on 7 August 2026) [38].

### 2.7. Prediction of Secondary Structure

The secondary structure of the vaccine construct was predicted using the GOR4 server (https://npsa.lyon.inserm.fr/cgi-bin/npsa_automat.pl?page=/NPSA/npsa_gor4.html, accessed on 8 August 2026). An additional prediction was performed using the PSIPRED server (http://bioinf.cs.ucl.ac.uk/psipred/, accessed on 8 August 2026) [39]. These analyses were used to predict the secondary structural features of the vaccine construct.

### 2.8. Modeling and Validation of Tertiary Structure

The finalized vaccine sequence was submitted to AlphaFold3 (https://alphafoldserver.com/, accessed on 8 August 2026) [40] to obtain a three-dimensional structural model. The initial model was then refined using the GalaxyRefine server (https://galaxy.seoklab.org/, accessed on 9 August 2026) to improve local structure and side-chain conformations before further analysis [41]. The refined structure was evaluated using several structure-validation methods. Stereochemical quality was assessed using the Ramachandran plot generated by the PROCHECK module of the UCLA-DOE LAB—SAVES v6.1 platform (https://saves.mbi.ucla.edu/, accessed on 10 August 2026) [42]. Structural quality was further assessed using ERRAT, which evaluates the reliability of a protein structure based on patterns of non-bonded atomic interactions [43]. An additional assessment was performed using the ProSA-web server (https://prosa.services.came.sbg.ac.at/prosa.php, accessed on 10 August 2026) [44], and the calculated Z-score was compared with scores reported for experimentally determined protein structures of similar size.

### 2.9. Docking with TLRs and Interaction Assessment

To investigate recognition of the vaccine construct by innate immune receptors, protein-protein docking analyses were performed against human TLR2 (PDB ID: 6NIG) and TLR4 (PDB ID: 3FXI) using the ClusPro 2.0 docking platform (https://cluspro.org/, accessed on 12 August 2026) [45]. TLR2 and TLR4 were selected as molecular docking targets because they are cell-surface Toll-like receptors involved in innate immune recognition and signaling. TLR2 primarily recognizes bacterial lipoproteins and lipopeptides, and its activation through TLR2-containing receptor complexes initiates intracellular signaling that promotes inflammatory responses and contributes to the development of adaptive immunity [46,47]. TLR4 functions as an important mediator of innate immune signaling, with ligand recognition activating MyD88-dependent and TRIF-dependent pathways that induce NF-κB, MAPKs, and interferon regulatory factors, leading to the production of inflammatory mediators and regulation of subsequent immune responses [47]. Therefore, TLR2 and TLR4 were selected to assess the potential interaction of the designed multi-epitope vaccine construct with these innate immune signaling pathways. For each receptor, the docking model with the most favorable ClusPro score was selected for structural analysis. The resulting vaccine–receptor complexes were then examined using PDBsum via the Tamarind Bio platform (https://app.tamarind.bio/tools/pdbsum, accessed on 14 August 2026) to characterize intermolecular contacts and identify the residues involved in complex formation [48]. Additional interface analysis was performed using COCOMAPS 2.0 (https://aocdweb.com/BioTools/cocomaps2, accessed on 14 August 2026) [49], providing complementary information on residue–residue contacts, interface composition, and other structural features involved in receptor recognition.

### 2.10. Molecular Dynamics Simulations

The structural stability of the vaccine complexes formed with TLR2 and TLR4 was further examined through molecular dynamics simulations performed in the Desmond simulation environment (Schrödinger, LLC, New York, NY, USA) [50]. Before initiating the simulations, each docked complex was prepared in Maestro using the Protein Preparation Wizard. During this procedure, bond orders were corrected, missing hydrogen atoms were added, protonation states appropriate for physiological pH were assigned, and hydrogen-bonding networks were optimized to improve structural consistency. Each prepared complex was then solvated in an orthorhombic periodic box containing the TIP3P explicit solvent model [51]. Counterions were added to neutralize the systems, followed by the addition of sodium chloride to achieve a physiological ionic concentration of 0.15 M. All simulations were performed using the OPLS-2005 force field [52]. Long-range electrostatic interactions were treated using the particle mesh Ewald (PME) approach, and periodic boundary conditions were applied throughout the simulations [53]. Following system equilibration, independent production trajectories of 200 ns were generated for each receptor–vaccine complex. The simulation trajectories were subsequently analyzed to characterize the dynamic behavior of the complexes over time. Multiple structural analysis, including root mean square deviation (RMSD), root mean square fluctuation (RMSF), radius of gyration (Rg), solvent-accessible surface area (SASA), and intermolecular hydrogen-bond occupancy, were calculated throughout the trajectories to assess structural flexibility, compactness, stability, and receptor–vaccine interactions. To further estimate the thermodynamic favorability of complex formation, binding free energies were calculated using the molecular mechanics generalized Born surface area (MM-GBSA) approach [54].

### 2.11. In Silico Cloning

To assess whether the vaccine construct could be produced recombinantly, an in silico expression strategy for *Escherichia coli* was performed. The protein sequence was first reverse-translated into a DNA coding sequence using the EMBOSS Backtranseq program (https://www.ebi.ac.uk/jdispatcher/st/emboss_backtranseq, accessed on 18 August 2026) [55]. The generated nucleotide sequence was then optimized using JCat (https://www.jcat.de/, accessed on 11 September 2026) for expression in *E. coli* K12 while preserving the encoded protein sequence [56]. The optimized coding sequence was subsequently analyzed using RNAfold (https://rna.tbi.univie.ac.at/, accessed on 18 August 2026) to examine the secondary structure of the transcript and its predicted folding behavior. For cloning, restriction recognition sites corresponding to NdeI and XhoI were introduced at the 5′ and 3′ termini of the optimized coding region, respectively. The resulting DNA fragment was inserted into the pET-28a(+) expression vector using SnapGene v8.2.1, producing the recombinant plasmid construct. The completed plasmid was finally examined to confirm the correct insert orientation, integration of the optimized coding sequence, and preservation of the intended cloning design before experimental expression studies were undertaken [57].

## 3. Results

### 3.1. Selection and Screening of Surface Proteins

A total of 41 surface-associated proteins were initially identified and subjected to the screening process (Supplementary Material S1). Four surface-associated proteins satisfied the predefined screening criteria and were selected for subsequent analyses (Table 1). The selected proteins included peptide ABC transporter substrate-binding protein (QCY38924.1), polysaccharide deacetylase (QCY37413.1), LPXTG cell wall anchor domain-containing protein (QCY37432.1), and YSIRK-type signal peptide-containing protein (QCY37300.1). All four proteins exhibited antigenicity scores above the VaxiJen v2.1 threshold of 0.4, ranging from 0.5087 to 0.8197, and were predicted to be non-allergenic. Among the selected proteins, the YSIRK-type signal peptide-containing protein had the highest antigenicity score (0.8197), whereas the polysaccharide deacetylase had the lowest score (0.5087). Subcellular localization analysis classified all four proteins as extracellular (Table 1). Signal peptide prediction identified secretion signals in each of the selected proteins (Figure 2). The peptide ABC transporter substrate-binding protein and polysaccharide deacetylase were predicted to contain Sec/SPII lipoprotein signal peptides, with cleavage sites located between amino acid positions 19–20 and 20–21, respectively. The LPXTG cell wall anchor domain-containing protein and the YSIRK-type signal peptide-containing protein were predicted to possess Sec/SPI signal peptides, with cleavage sites between residues 29–30 and 35–36, respectively (Figure 2).

Transmembrane topology analysis showed differences in the membrane organization of the selected proteins (Figure 3). According to the predefined screening criteria, proteins containing 0–1 predicted transmembrane helix were retained for further analyses. The peptide ABC transporter substrate-binding protein and polysaccharide deacetylase did not contain any predicted transmembrane helices and were predominantly localized on the extracellular side of the membrane. In contrast, the LPXTG cell wall anchor domain-containing protein and the YSIRK-type signal peptide-containing protein each contained one predicted transmembrane helix. The transmembrane helix of the LPXTG cell wall anchor domain-containing protein was located between amino acid residues 285–304, with the remaining regions predicted as extracellular (1–284) and intracellular (305–310). The YSIRK-type signal peptide-containing protein contained a transmembrane helix between residues 13–35, with the remaining regions predicted as intracellular (1–12) and extracellular (36–749). Thus, two selected proteins had no predicted transmembrane helices, whereas the other two contained a single transmembrane helix each, with the major portions of both proteins predicted to be extracellular. The selected epitopes were located outside the predicted transmembrane regions and therefore did not overlap with the identified transmembrane helices.

The sequence conservation of the four selected surface proteins was evaluated using Shannon entropy analysis based on homologous protein sequences exhibiting more than 90% sequence identity and 90% query coverage (Figure 4). Distinct entropy profiles were observed among the analyzed proteins, indicating differences in residue variability across their respective sequences. The peptide ABC transporter substrate-binding protein exhibited relatively low entropy values throughout most of its sequence, with only a limited number of positions displaying increased variability (Figure 4A). The polysaccharide deacetylase showed comparatively higher entropy values across multiple regions of the protein sequence, although several conserved regions with lower entropy values were also present (Figure 4B). The LPXTG cell wall anchor domain-containing protein showed predominantly low entropy values, with discrete peaks at specific residue positions, indicating localized sequence variation (Figure 4C). In contrast, the YSIRK-type signal peptide-containing protein displayed greater variability in the C-terminal portion of the sequence, whereas the N-terminal region remained comparatively less variable (Figure 4D). Overall, the entropy analysis identified both conserved and variable regions within the selected proteins, providing a basis for subsequent epitope conservancy assessment.

### 3.2. Identification of Epitopes

B-cell epitope mapping identified multiple candidate epitopes from the selected surface proteins. A total of eight linear (continuous) B-cell epitopes were initially predicted across the four selected proteins, including one epitope from the peptide ABC transporter substrate-binding protein, four from the polysaccharide deacetylase, one from the LPXTG cell wall anchor domain-containing protein, and two from the YSIRK-type signal peptide-containing protein.

These candidate peptides were evaluated based on epitope length, antigenicity, allergenicity, and toxicity. Epitopes located within the predicted transmembrane regions were also excluded from further consideration. The BepiPred 3.0 prediction scores were not used to rank the candidate epitopes. Following this filtering process, three epitopes were identified as antigenic, non-allergenic, and non-toxic and were retained for subsequent analyses (Table 2). These included the epitope RETGKKRK from the peptide ABC transporter substrate-binding protein and the epitopes NTSSNEINP and NTQNSDNS from the YSIRK-type signal peptide-containing protein. Each retained epitope had an antigenicity value above the established cutoff and was classified as non-allergenic and non-toxic. Of the four epitopes predicted from the polysaccharide deacetylase, one was 31 amino acids long and was excluded, whereas the remaining three were 6 amino acids long and were predicted to be allergenic. The single epitope predicted from the LPXTG cell wall anchor domain-containing protein was 243 amino acids long and was excluded during filtering.

Candidate CTL epitopes from the selected surface proteins were further evaluated for antigenicity, allergenicity, toxicity, and predicted HLA class I binding. Following this screening, six CTL epitopes met the predefined selection criteria and were retained for subsequent analyses (Table 3). These included DQQAGASLY and TADDFVYAW from the peptide ABC transporter substrate-binding protein, VAIDGLNLY and SVFTVLIAI from the polysaccharide deacetylase, SQTSLHHEL from the LPXTG cell wall anchor domain-containing protein, and RQKSDLNLF from the YSIRK-type signal peptide-containing protein. The retained CTL epitopes had antigenicity values ranging from 0.5510 to 1.1271 and were predicted to be non-allergenic and non-toxic. HLA class I binding analysis predicted interactions between each epitope and multiple HLA class I alleles, although the number of predicted HLA-binding alleles varied among the epitopes (Table 3).

HTL epitope prediction followed by immunological screening identified four epitopes that met the predefined selection criteria (Table 4). The selected peptides included VNKYKNSKALNHRLT from the peptide ABC transporter substrate-binding protein, VEYKGDVAHIFFHPL from the polysaccharide deacetylase, QNGLVKQTSKSNSQQ from the LPXTG cell wall anchor domain-containing protein, and TVDYGAAHAAKYGHE from the YSIRK-type signal peptide-containing protein. The retained HTL epitopes had antigenicity values ranging from 0.6825 to 0.8897 and were predicted to be non-allergenic and non-toxic. The selected HTL peptides also showed predicted interactions with multiple HLA class II alleles, with the number of predicted allele interactions varying among the individual peptides (Table 4).

Sequence homology analysis showed that none of the 13 selected epitopes exhibited complete sequence similarity with the corresponding human protein sequences. The identified matches had alignment lengths ranging from 5 to 12 amino acids. Percentage identity ranged from 63.64% to 100%, whereas E-values ranged from 1.4 to 46, with all identified E-values greater than 1. Four epitopes, NTSSNEINP, TADDFVYAW, VAIDGLNLY, and SQTSLHHEL, showed 100% identity within their respective aligned regions. However, these 100% identity values corresponded only to specific regions of the respective epitopes, involving 7, 5, 7, and 6 aligned amino acids, respectively, rather than the complete epitope sequences. The corresponding E-values for these matches were 1.7, 14, 2.4, and 9.8, respectively. The remaining epitopes showed partial sequence identities ranging from 63.64% to 87.50% within their respective aligned regions. The complete BLASTp results, including the human protein names, matching protein accessions, alignment lengths, percentage identities, and E-values, are provided in Supplementary Material S2.

Overall, none of the identified matches met the predefined E-value threshold of ≤0.001 for significant sequence homology with human proteins. Thus, no significant sequence homology with human proteins was identified among the selected epitopes based on this criterion. However, these findings are based on computational sequence analysis and cannot definitively exclude cross-reactivity or molecular mimicry. Experimental validation will therefore be required to assess potential cross-reactivity and the immunological safety of the selected epitopes.

### 3.3. Epitope Conservancy Analysis

The sequence conservancy of the shortlisted epitopes was evaluated across 30 homologous protein sequences for each of the four selected target proteins. The results of the epitope conservancy analysis of the selected epitopes are provided in Supplementary Material S3. For the peptide ABC transporter substrate-binding protein, the HTL epitope VNKYKNSKALNHRLT exhibited 100% sequence conservancy across all analyzed homologs. The CTL epitopes DQQAGASLY and TADDFVYAW also showed high sequence conservation, with DQQAGASLY exhibiting 100% conservancy in 29 homologous sequences and 88.89% conservancy in one homolog, while TADDFVYAW remained 100% conserved in 29 homologs and showed 88.89% conservancy in one sequence. In contrast, the B-cell epitope RETGKKRK showed greater sequence variation, exhibiting 100% conservancy in six homologs, 87.50% conservancy in two homologs, and 50% conservancy in the remaining 22 homologous sequences.

Similarly, the shortlisted epitopes identified in the polysaccharide deacetylase showed extensive sequence conservation among the examined homologs. The HTL epitope VEYKGDVAHIFFHPL remained 100% conserved across all 30 homologous sequences. Among the CTL epitopes, VAIDGLNLY exhibited 100% conservancy in 28 homologs and 88.89% conservancy in two homologous sequences, whereas SVFTVLIAI remained 100% conserved in 29 homologs, with only one sequence exhibiting 88.89% conservancy.

For the LPXTG cell wall anchor domain-containing protein, both shortlisted epitopes showed complete sequence conservation across all analyzed homologs. The HTL epitope QNGLVKQTSKSNSQQ and the CTL epitope SQTSLHHEL each exhibited 100% conservancy in all 30 homologous protein sequences, with no sequence variation detected among the evaluated homologs.

Likewise, the shortlisted epitopes derived from the YSIRK-type signal peptide-containing protein exhibited high levels of sequence conservation. The HTL epitope TVDYGAAHAAKYGHE remained 100% conserved across all analyzed homologs. The CTL epitope RQKSDLNLF exhibited 100% conservancy in 23 homologous sequences, whereas the remaining seven homologs showed 88.89% conservancy. Among the B-cell epitopes, NTSSNEINP remained 100% conserved in 29 homologous sequences, with only one homolog exhibiting 88.89% conservancy. In comparison, NTQNSDNS displayed greater sequence variation, exhibiting 100% conservancy in 10 homologous sequences, 87.50% conservancy in 17 homologs, and 75% conservancy in three homologous sequences.

### 3.4. Assessment of Population Coverage

The population coverage results showed an estimated global coverage of 97.50% for the selected CTL and HTL epitopes. Regional population coverage varied from 86.45% in South America to 98.84% in Europe. Coverage values greater than 98% were observed in Europe (98.84%), North America (98.58%), East Asia (98.31%), and the West Indies (98.12%). Population coverage exceeding 90% was predicted for West Africa (95.60%), North Africa (96.11%), East Africa (94.35%), South Asia (91.69%), Southeast Asia (91.89%), and Oceania (90.79%). The remaining regions, including Central Africa (89.90%), Northeast Asia (88.87%), Southwest Asia (87.79%), South Africa (86.86%), and South America (86.45%), also showed predicted population coverage above 85%. Overall, the selected CTL and HTL epitopes showed high predicted population coverage across the evaluated geographical regions, with regional variation in the estimated coverage values (Figure 5).

### 3.5. Vaccine Construct Design

The final vaccine construct consisted of 254 amino acids and incorporated three B-cell, six CTL, and four HTL epitopes. Human β-defensin-3 was positioned at the N-terminus as an immunostimulatory adjuvant and was connected to the epitope region through an EAAAK linker. A PADRE peptide was positioned near the C-terminus after the last B-cell epitope and connected through an additional EAAAK linker. A C-terminal 6×His tag was included to facilitate purification of the recombinant protein. The final vaccine construct showed a VaxiJen antigenicity score of 0.8609, which was above the VaxiJen cutoff of 0.4, and was predicted to be non-allergenic. Based on the predefined computational screening criteria, the construct was selected for subsequent structural and immunological analyses (Figure 6).

### 3.6. Physicochemical Property Analysis

The physicochemical properties of the finalized multi-epitope vaccine construct were evaluated using ProtParam, while SoluProt was used to predict recombinant protein solubility. The construct consisted of 254 amino acid residues, with a calculated molecular weight of 27,578.17 Da and a theoretical pI of 9.75. The calculated instability index was 22.72, while the aliphatic index and grand average of hydropathicity (GRAVY) were 64.33 and −0.665, respectively. The predicted solubility score was 0.862. These calculated physicochemical properties are summarized in Table 5.

### 3.7. Prediction of Secondary Structure

The secondary structure of the vaccine construct was predicted using GOR4 and PSIPRED. According to the GOR4 analysis, the vaccine construct contained 106 α-helical residues, representing 41.73% of the sequence, 39 extended strand residues, representing 15.35%, and 109 random coil residues, accounting for 42.91% of the total 254 amino acids (Figure 7A). The predicted secondary structure therefore consisted mainly of α-helices and random coils, while extended strands represented a smaller proportion of the sequence. The PSIPRED analysis provided a residue-level representation of the predicted secondary structure, showing the distribution of α-helices, β-strands, and coil regions across the complete 254-residue sequence (Figure 7B).

### 3.8. Modeling of Tertiary Structure and Validation

The initial three-dimensional model of the vaccine construct was generated using AlphaFold3 and used as the starting structure for subsequent refinement and validation (Figure 8A). Structural refinement improved the distribution of residues in the Ramachandran plot. Before refinement, 82.6% of amino acid residues were located in the most favored regions, while 17.4% were distributed in additionally allowed regions. No residues were located in generously allowed or disallowed regions. Following refinement, the proportion of residues in the most favored regions increased to 98.6%, while the remaining 1.4% were located exclusively in additionally allowed regions. No residues were present in the generously allowed or disallowed regions after refinement (Figure 8B). Further structural validation showed an ERRAT overall quality factor of 88.89, indicating the quality of the refined model based on the distribution of non-bonded interactions (Figure 8D). ProSA-web analysis of the refined structure produced a Z-score of −3.85. This value was within the range observed for experimentally determined protein structures of comparable size, as shown in the ProSA-web quality assessment (Figure 8C).

### 3.9. Docking with TLRs and Interaction Assessment

The molecular docking analysis showed that the vaccine–TLR2 complex exhibited a weighted docking score of −1136.9 with 45 members, whereas the vaccine–TLR4 complex exhibited a weighted docking score of −1195.0 with 43 members. For both complexes, Cluster 0 was selected for interaction analysis based on its larger cluster size. The residue-level interactions involving the designed multi-epitope vaccine construct and TLR2 were characterized to examine the predicted receptor–vaccine interface. The docked complex contained 17 hydrogen bonds, 5 salt bridges, and 190 non-bonded contacts. Overall, 27 interface residues were identified in the docked complex, with interface surface areas of 1422 Å^2^ for TLR2 (chain D) and 1508 Å^2^ for the vaccine construct (chain V) (Figure 9). Analysis of the hydrogen-bonding network identified multiple interacting residues on both binding partners. The hydrogen-bond-forming residues of TLR2 included Ser243, Glu241, Phe239, Ser240, Asp299, Arg302, Ile304, Arg296, Asp301, Phe295, Asp327, Ser329, and Val292. The corresponding hydrogen-bond-forming residues of the vaccine construct comprised Lys207, Arg208, Thr204, Lys200, Lys201, Lys237, Asp77, Leu70, Trp242, Lys245, Leu244, and Asp187. These residue-to-residue interactions formed the hydrogen-bonding network across the predicted receptor–vaccine interface. In addition to hydrogen bonds, the interface also displayed several salt bridges and numerous non-bonded contacts, contributing to the overall intermolecular contact profile of the docked complex.

The residue-level interactions between the vaccine construct and TLR4 were examined to characterize the predicted receptor–vaccine binding interface. Docking analysis identified 15 hydrogen bonds, 5 salt bridges, and 210 non-bonded contacts within the complex. Interface mapping further showed that 27 residues participated in binding on the TLR4 molecule (chain B), whereas 29 residues contributed from the vaccine construct (chain V). The corresponding interface surface areas measured 1410 Å^2^ for TLR4 and 1454 Å^2^ for the vaccine construct (Figure 10). Analysis of the hydrogen-bonding network identified multiple interacting residues on both binding partners. The hydrogen-bond-forming residues of TLR4 included Ser589, Asp596, Gln588, Glu586, Asn624, Lys615, Asp614, Arg606, Glu605, Glu608, Gln578, and Lys561. The corresponding hydrogen-bond-forming residues of the vaccine construct comprised Leu106, Tyr110, Ser111, Tyr81, Tyr86, Thr75, Tyr71, Tyr74, Tyr59, His253, His250, and Asp103. These residue-to-residue hydrogen-bond interactions were distributed across the predicted receptor–vaccine interface, contributing to the intermolecular contact network.

The intermolecular interfaces formed between the vaccine construct and TLR2 or TLR4 were independently compared using COCOMAPS server to assess their respective contact characteristics. The TLR2-bound complex involved 71 contacting residues, consisting of 39 residues from the receptor and 32 from the vaccine construct. In comparison, the TLR4-bound complex comprised 66 contact-forming residues, including 34 receptor residues and 32 vaccine residues. The overall buried interface measured 2930.2 Å^2^ for the TLR2 complex and 2873.9 Å^2^ for the TLR4 complex. The corresponding interface areas were 1465.1 Å^2^ and 1436.95 Å^2^, respectively. The TLR2 complex exhibited a polar interface area of 514.65 Å^2^ (35.13%) and a non-polar interface area of 950.5 Å^2^ (64.88%), while the corresponding values for the TLR4 complex were 541.75 Å^2^ (37.70%) and 895.2 Å^2^ (62.30%), respectively. Comparison of the atomic interactions showed differences in the intermolecular contact profiles between the two receptor–vaccine complexes. The vaccine–TLR2 complex contained 48 proximal contacts, 19 CH–O/N bonds, 12 hydrogen bonds, 8 apolar van der Waals contacts, 5 salt bridges, 3 steric clashes, 1 π–π interaction, and 1 CH–π interaction. In contrast, the vaccine–TLR4 complex exhibited 60 proximal contacts, 20 CH–O/N bonds, 6 hydrogen bonds, 18 apolar van der Waals contacts, 3 salt bridges, 6 steric clashes, 4 CH–π interactions, and 1 lp–π interaction. The relative distribution of the intermolecular contact types for both receptor–vaccine complexes is illustrated in Figure 11, while the quantitative interface parameters are summarized in Table 6.

### 3.10. Molecular Dynamics Simulations

The dynamic behavior of the receptor–vaccine systems during the 200 ns simulations was characterized using RMSD as the primary stability metric. In the vaccine–TLR2 system, RMSD values increased rapidly from approximately 7.0 Å at the beginning of the simulation to nearly 9.0 Å within the first 10 ns. A gradual increase was then observed, reaching a maximum of approximately 11.0 Å between 45 and 55 ns. Thereafter, the RMSD decreased slightly and fluctuated predominantly between 9.5 and 10.5 Å for the remainder of the simulation, with no additional large deviations observed after approximately 100 ns (Figure 12A). The individual RMSD plots showed distinct patterns for the receptor and vaccine construct. The TLR2 receptor (Protein A) exhibited comparatively lower RMSD values, remaining predominantly within the range of approximately 2.5–4.0 Å throughout the simulation, with limited fluctuations. In contrast, the vaccine construct (Protein B) showed higher RMSD values, increasing from approximately 13.0 Å at the beginning of the simulation to approximately 16.5–17.0 Å during the first 50 ns. Subsequently, the RMSD fluctuated mainly between 14.5 and 16.0 Å (Figure 12B).

In the vaccine–TLR4 complex, RMSD values increased from approximately 6.0 Å to nearly 8.0 Å during the first 10 ns of the simulation. The values continued to increase gradually, reaching approximately 8.8 Å around 50–60 ns. A transient decrease to approximately 7.8 Å was observed between 70 and 80 ns, after which the RMSD returned to values between 8.2 and 8.8 Å and remained within this range until the end of the MD trajectory. Compared with the TLR2-bound complex, the TLR4-bound system maintained a tighter RMSD distribution during the later portion of the molecular dynamics trajectory (Figure 12C). Analysis of the individual RMSD plots indicated that the TLR4 receptor (Protein A) remained predominantly between approximately 2.5 and 5.0 Å, with moderate fluctuations throughout the simulation. The vaccine construct (Protein B) increased rapidly to approximately 13.0 Å within the initial 10 ns and thereafter fluctuated between approximately 12.5 and 13.8 Å throughout the remainder of the simulation (Figure 12D).

Residue-specific flexibility across the receptor–vaccine complexes was assessed from the 200 ns molecular dynamics trajectories using RMSF. In the vaccine–TLR2 system, TLR2 encompassed residues 1–549, whereas the corresponding vaccine segment included residues 550–803. The receptor region showed relatively smaller residue fluctuations across most of the sequence, with RMSF values generally ranging from approximately 1.0 to 3.0 Å. Several localized peaks reaching approximately 3.0–3.5 Å were observed within the receptor region, while residue fluctuations gradually increased toward the receptor–vaccine junction. In contrast, the vaccine region exhibited comparatively higher RMSF values, with most residues fluctuating between approximately 2.0 and 5.0 Å. Prominent peaks were observed between approximately residues 670 and 730, where RMSF values approached 10.0 Å, followed by lower values and moderate fluctuations toward the C-terminus (Figure 13A).

In the vaccine–TLR4 complex, residues 1–601 corresponded to TLR4, whereas residues 602–855 represented the vaccine construct. The receptor region displayed RMSF values predominantly ranging from approximately 1.0 to 3.0 Å, with relatively small fluctuations across most residues and a gradual increase toward the receptor–vaccine interface. The vaccine construct exhibited comparatively higher RMSF values than the receptor, with most residues fluctuating between approximately 2.0 and 6.0 Å. Distinct peaks were observed between approximately residues 730 and 820, where RMSF values approached 9.0 Å, followed by moderate fluctuations toward the terminal residues (Figure 13B).

The radius of gyration (Rg) and SASA profiles were recorded for both receptor–vaccine systems over the 200 ns simulations. The trajectories were analyzed to assess changes in the structural properties of each complex throughout the simulations. For the vaccine–TLR2 system, the Rg gradually increased during the first 20 ns, reaching approximately 32.8 Å, followed by fluctuations predominantly between 31.0 and 32.0 Å for most of the simulation. A slight decrease was observed during the final 20 ns, when the trajectory approached approximately 30.8–31.2 Å (Figure 14A). The vaccine–TLR4 complex exhibited Rg values ranging from approximately 34.5 to 38.0 Å. Its trajectory decreased rapidly during the early phase of the simulation from nearly 38.0 Å to 35.5 Å, after which it fluctuated mainly between 35.0 and 36.2 Å over the remaining simulation period, with occasional increases approaching 37.0 Å (Figure 14B).

The SASA trajectory for the vaccine–TLR2 system began at approximately 45,500 Å^2^, followed by a rapid decrease during the first 20 ns to approximately 40,000 Å^2^. Subsequently, the SASA gradually declined and fluctuated predominantly between 36,500 and 39,000 Å^2^ until the completion of the simulation, with moderate variations observed during the latter half of the trajectory (Figure 14C). Likewise, the vaccine–TLR4 system displayed an initial SASA value of approximately 46,500 Å^2^, which decreased rapidly to around 40,000 Å^2^ within the first 20 ns. Thereafter, the trajectory remained largely between 38,000 and 40,000 Å^2^ throughout the remaining simulation period, with moderate variability observed throughout the 200 ns trajectory (Figure 14D).

The evolution of secondary structural elements was evaluated for both receptor–vaccine complexes during the 200 ns simulations. For the TLR2-associated vaccine complex, the overall secondary structure content remained close to 28–30% throughout the simulation, with only minor variations over time (Figure 15A). The residue-level secondary structural profile indicated that most structural elements were retained throughout the simulation, while localized changes were observed primarily within loop and terminal regions. The receptor region showed comparatively consistent secondary structural elements, whereas several segments of the vaccine construct exhibited intermittent transitions between ordered and coil conformations.

Similarly, the vaccine–TLR4 complex maintained an overall secondary structure content of approximately 28–30% during the 200 ns simulation, with only small fluctuations throughout the analysis (Figure 15B). The residue-level secondary structural pattern indicated that the majority of structural elements remained preserved throughout the simulation period. Minor localized changes were observed in selected residue segments, predominantly within the vaccine region, whereas the receptor-associated secondary structural elements showed comparatively fewer variations.

The PCA of the 200 ns trajectory was performed to assess the major collective motions of the TLR2-bound vaccine system. The first three principal components, PC1, PC2, and PC3, explained 32.94%, 15.65%, and 10.04% of the total conformational variance, respectively, with a cumulative contribution of 58.63% (Figure 16). The PC1–PC2, PC2–PC3, and PC1–PC3 projections showed that the trajectory sampled multiple conformational regions, with a gradual transition from the initial (red) to the later (blue) simulation frames. Partial overlap among the conformational clusters was observed throughout the trajectory. The scree plot showed a progressive decrease in the contribution of successive principal components, with the cumulative variance reaching approximately 65.0%, 77.3%, and 88.3% after the first four, seven, and twenty principal components, respectively. These results indicate that the leading principal components accounted for a substantial proportion of the observed conformational variance.

The PCA of the TLR4-bound vaccine system was performed to assess the major collective motions of the complex. The first three principal components, PC1, PC2, and PC3, accounted for 26.71%, 14.22%, and 10.53% of the total structural variance, respectively, with a cumulative contribution of 51.46% (Figure 17). The PC1–PC2, PC2–PC3, and PC1–PC3 projections showed that the system sampled several conformational states as the simulation progressed, with a gradual shift from the early (red) to the later (blue) simulation frames. Partial overlap among the conformational clusters was observed throughout the trajectory. The scree plot showed a steady decrease in the variance explained by successive principal components. The first four, seven, and twenty principal components accounted for approximately 60.5%, 73.8%, and 86.4% of the cumulative variance, respectively, indicating that the leading principal components accounted for a substantial proportion of the observed conformational variance.

The correlated atomic motions in the receptor–vaccine complexes were evaluated using DCCM over the 200 ns MD trajectories. In the vaccine–TLR2 complex, the DCCM showed extensive regions of positively correlated motions distributed within both the receptor and vaccine regions, together with correlated movements extending across the receptor–vaccine interface (Figure 18A). These positively correlated regions were primarily concentrated along the main diagonal of the matrix, indicating coordinated motions among neighboring residues. In addition, localized regions of anti-correlated motions were observed throughout the matrix, although their distribution was comparatively limited relative to the positively correlated regions. Similarly, the DCCM of the vaccine–TLR4 complex showed widespread positively correlated residue motions across both the receptor and vaccine regions (Figure 18B). Correlated motions were also observed between residues located at the receptor–vaccine interface, indicating coordinated movements between the interacting partners during the simulation. Anti-correlated motions appeared as smaller and scattered regions throughout the matrix, whereas the majority of residue pairs exhibited positive or weakly correlated motions. Overall, the DCCM analyses showed coordinated residue motions in both receptor–vaccine complexes.

The hydrogen-bond occupancy and total interaction energy were monitored for both receptor–vaccine systems over the 200 ns MD trajectories. In the vaccine–TLR2 system, the number of hydrogen bonds increased from approximately 8–12 at the start of the simulation to nearly 18–22 within the first 30–40 ns. Thereafter, the values fluctuated predominantly between 18 and 24, with occasional increases approaching 28 intermolecular hydrogen bonds during the later phase of the simulation (Figure 19A). Likewise, the vaccine–TLR4 complex exhibited an initial increase in the number of hydrogen bonds, rising from approximately 4–8 to around 18–22 during the first 20–30 ns. Throughout the remainder of the simulation, the total number of hydrogen bonds fluctuated mainly between 15 and 23, with occasional transient increases reaching approximately 27–29 hydrogen bonds (Figure 19B).

The vaccine–TLR2 system exhibited an initial decrease in total interaction energy to approximately −16,100 kcal/mol. Following this initial change, the total energy fluctuated predominantly between approximately −14,500 and −12,800 kcal/mol over the remaining simulation period, with moderate variations throughout the trajectory (Figure 19C). For the vaccine–TLR4 complex, the total energy initially decreased to approximately −15,200 kcal/mol, followed by an increase during the first 20–30 ns. Subsequently, the values fluctuated mainly between approximately −14,800 and −13,300 kcal/mol, with occasional transient decreases approaching −15,500 kcal/mol during the latter part of the simulation (Figure 19D).

Binding free-energy calculations and the complete energy decomposition obtained from the final frame of the 200 ns molecular dynamics simulations are summarized in Table 7. The vaccine–TLR4 complex exhibited a more favorable predicted binding free energy (ΔG_bind = −255.343 kcal/mol) than the vaccine–TLR2 complex (ΔG_bind = −9.04983 kcal/mol). A marked difference was observed in the Coulombic contribution, which was positive for the vaccine–TLR2 system (468.7526 kcal/mol) but negative for the vaccine–TLR4 system (−339.180 kcal/mol). The other energy components, including covalent, hydrogen-bond, lipophilic, packing, self-contact, Solv_GB, Solv_SA, and van der Waals contributions, are presented in Table 7.

The raw data generated and analyzed during this study are provided in Supplementary Material S4.

### 3.11. In Silico Cloning

The vacxcine sequence was evaluated for its suitability for recombinant expression in *Escherichia coli* K12 through codon optimization. Before optimization, the sequence showed a codon adaptation index (CAI) of 0.576 and a GC content of 57.09%. Following optimization, the CAI increased to 1.000, while the GC content decreased to 50.79%. These changes resulted in a codon usage profile with a higher predicted adaptation to the selected bacterial host and a GC content within the optimized range. The predicted secondary structures of the optimized mRNA generated by RNAfold are shown in Figure 20.

Evaluation of the optimized nucleotide sequence yielded a minimum free-energy (MFE) structure with an energy value of −224.10 kcal/mol. The corresponding thermodynamic ensemble was estimated at −237.90 kcal/mol. The centroid model had a predicted energy of −124.63 kcal/mol, while the calculated ensemble diversity was 199.14. Both the MFE and centroid structures consisted of multiple stem-loop and hairpin regions distributed throughout the mRNA sequence, reflecting the predicted secondary structural organization of the optimized transcript. Following sequence optimization, the vaccine coding region was computationally inserted into the pET-28a(+) bacterial expression plasmid for in silico cloning. The resulting recombinant construct placed the insert downstream of the T7 promoter while preserving the intended translational reading frame with the vector-derived 6×His affinity tag. The computational cloning result showed the incorporation of the optimized vaccine coding sequence into the selected expression plasmid (Figure 21).

## 4. Discussion

*S. haemolyticus* infections are particularly challenging to manage because antimicrobial resistance limits the effectiveness of available treatment options, while biofilm formation can restrict antimicrobial penetration and protect bacterial cells from host immune responses. Consequently, reverse vaccinology integrated with immunoinformatics has become a useful strategy for identifying conserved antigenic targets suitable for designing novel vaccine candidates against clinically significant bacterial species [58]. In the present investigation, this computational approach was applied to screen conserved surface-associated proteins for the construction of a multi-epitope vaccine candidate that showed favorable predicted immunological, physicochemical, and structural properties.

Selection of appropriate vaccine targets is fundamental to the development of epitope-based vaccines. Surface-exposed proteins were prioritized because they are readily accessible to immune surveillance and participate in bacterial adhesion and colonization. Similar target selection strategies have been applied for *Staphylococcus epidermidis*, where extracellular proteins with high antigenicity and low similarity to human proteins were identified as promising vaccine candidates to reduce the potential risk of host cross-reactivity while enhancing immune recognition [59]. Likewise, the high sequence conservation observed among the selected proteins in the present study supports their suitability for the development of vaccine candidates with the potential for broad strain coverage among genetically diverse *S. haemolyticus* isolates.

Both humoral and cellular immune responses can contribute to host defense against bacterial infections such as those caused by *S. haemolyticus*. B-cell epitopes were therefore included in the present construct to support the induction of antigen-specific antibodies, which may contribute to recognition and clearance of extracellular bacteria and their surface-associated components. CTL and HTL epitopes were incorporated to support cellular immune responses, while HTL responses can also contribute to the activation and coordination of other immune cells and support antibody production. Thus, the combined inclusion of B-cell, CTL, and HTL epitopes was intended to target complementary components of the adaptive immune response rather than relying on a single type of immune response.

Sequence similarity between vaccine epitopes and host proteins is another consideration during vaccine design, as shared sequences may potentially contribute to autoimmune reactions through molecular mimicry [60]. Therefore, human-proteome homology screening was incorporated into the present study to identify potential similarities between the selected epitopes and human proteins. Although no significant sequence homology was identified based on the applied BLASTp criterion, computational screening alone cannot exclude potential cross-reactivity, and experimental validation remains necessary to establish the safety of the selected epitopes.

The estimated global population coverage of 97.5% indicates that the selected epitopes are predicted to provide extensive representation across genetically diverse human populations. This finding supports the potential applicability of the proposed vaccine across individuals carrying a broad range of HLA alleles. Similar findings have also been described for a multi-epitope vaccine targeting multidrug-resistant *Enterococcus faecium*, where the inclusion of broadly recognized T-cell epitopes increased predicted population coverage and highlighted the importance of accounting for HLA diversity when designing vaccine candidates intended for widespread application [61].

The favorable physicochemical profile of the vaccine construct, including predicted antigenicity, lack of allergenic potential, predicted solubility, and acceptable structural properties, supports its further evaluation for recombinant expression and experimental validation. To support vaccine design, linker molecules and immunostimulatory components were incorporated to maintain separation between individual epitopes and to support appropriate antigen processing and presentation. A comparable design approach has been described for epitope-based vaccine development against *Streptococcus pneumoniae*, where optimization of the vaccine construct was associated with favorable predicted immunogenic and structural properties of the final construct [62].

Three-dimensional model construction followed by structural refinement further supported the quality of the proposed vaccine candidate. Structural assessment showed that most residues were located within the favored regions of the Ramachandran plot. Additional validation using ERRAT and ProSA plot further supported the quality of the refined model, with the results indicating favorable stereochemical properties and structural characteristics. Collectively, these findings indicate that the refined model is suitable for further computational investigations. Comparable improvements after structural optimization have also been described for a multi-epitope mRNA vaccine targeting *Streptococcus agalactiae*, where refinement improved the stereochemical properties of the predicted protein structure before molecular docking analyses [63].

Molecular docking combined with molecular dynamics analyses showed favorable predicted interactions between the vaccine candidate and Toll-like receptors, supporting their potential recognition by these innate immune receptors. The combination of favorable receptor interactions and the observed structural behavior throughout the simulations provides computational support for further investigation of the vaccine candidate. Comparable observations have been described for a multi-epitope vaccine targeting *Klebsiella pneumoniae*, where persistent receptor interactions and favorable dynamic behavior supported the predicted potential of the candidate before experimental validation [64]. Nevertheless, the present study has several limitations. The findings are based entirely on computational analyses, and the predicted antigenicity, structural properties, receptor interactions, and molecular dynamics behavior have not yet been experimentally validated. The immunogenicity, safety, expression, and protective efficacy of the vaccine candidate therefore remain to be established. Future studies should first confirm recombinant expression and purification of the vaccine construct, followed by in vitro immunogenicity and safety assessment. Subsequent animal studies should evaluate antibody and cellular immune responses, followed by challenge experiments to determine whether vaccination provides protection against *S. haemolyticus* infection. These experimental studies will be essential to determine whether the computationally predicted properties of the candidate translate into measurable immunological responses and protective efficacy.

## 5. Conclusions

Using an integrated immunoinformatics approach, this study identified conserved antigenic regions from multiple surface-associated proteins to construct and evaluate a candidate multi-epitope vaccine against *S. haemolyticus*. The resulting construct showed favorable predicted immunological and structural properties, extensive predicted HLA coverage across diverse populations, and molecular features compatible with further evaluation for recombinant protein production, supporting its potential as a candidate for further experimental evaluation against multidrug-resistant *S. haemolyticus* infections. The use of conserved surface antigens may also support immune recognition across genetically variable strains while reducing the potential impact of antigenic sequence variation. Although these computational findings are encouraging, they require experimental validation before their biological and protective relevance can be established. Future work should focus on recombinant production of the proposed vaccine antigen, followed by experimental characterization, systematic evaluation of immune responses, validation of protective efficacy in relevant animal models, and detailed assessment of safety and long-term immune persistence before progression toward preclinical development.

## Figures and Tables

**Figure 1:**
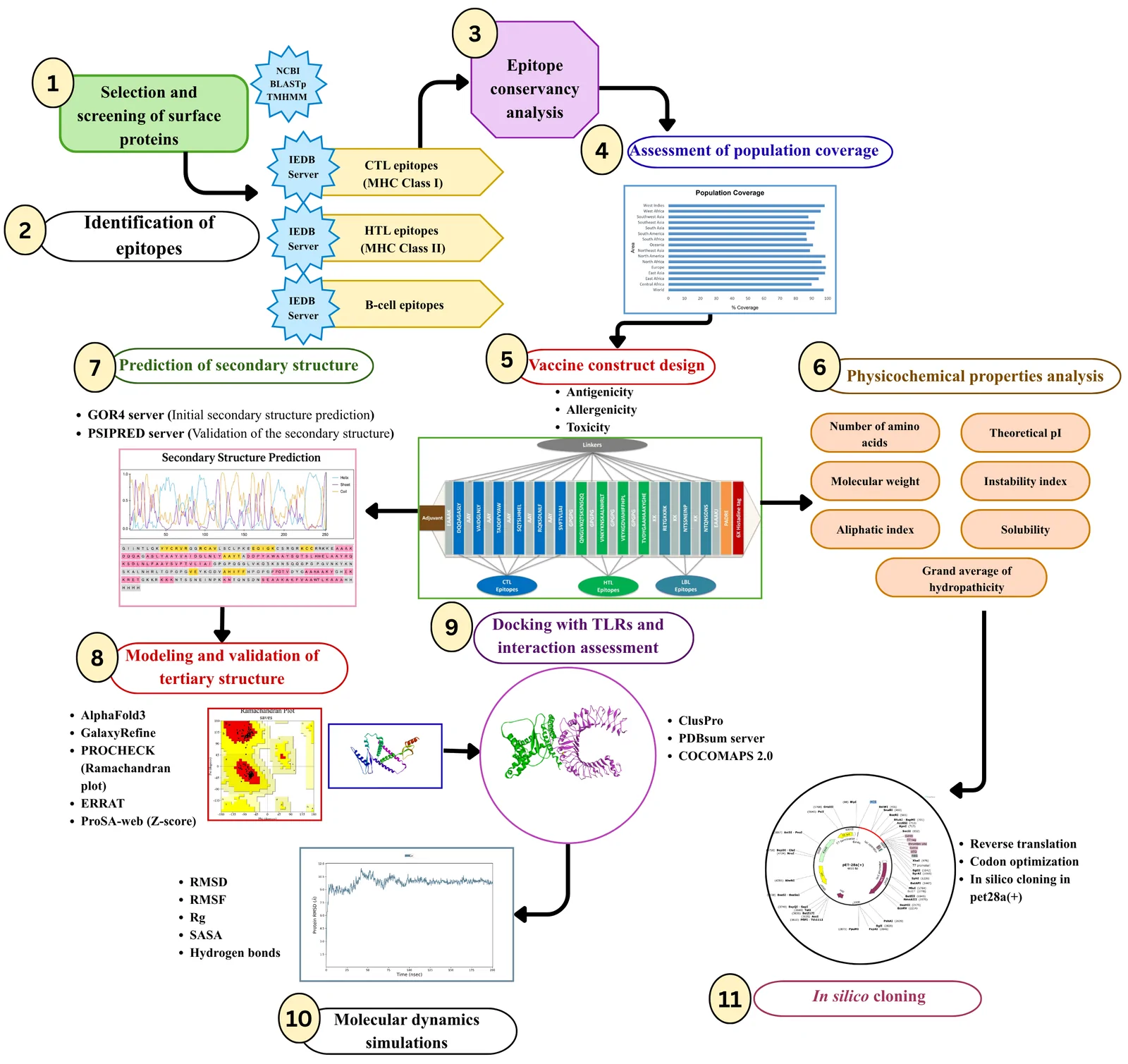
Computational pipeline adopted to identify, assemble, and assess the designed multi-epitope vaccine.

**Figure 2:**
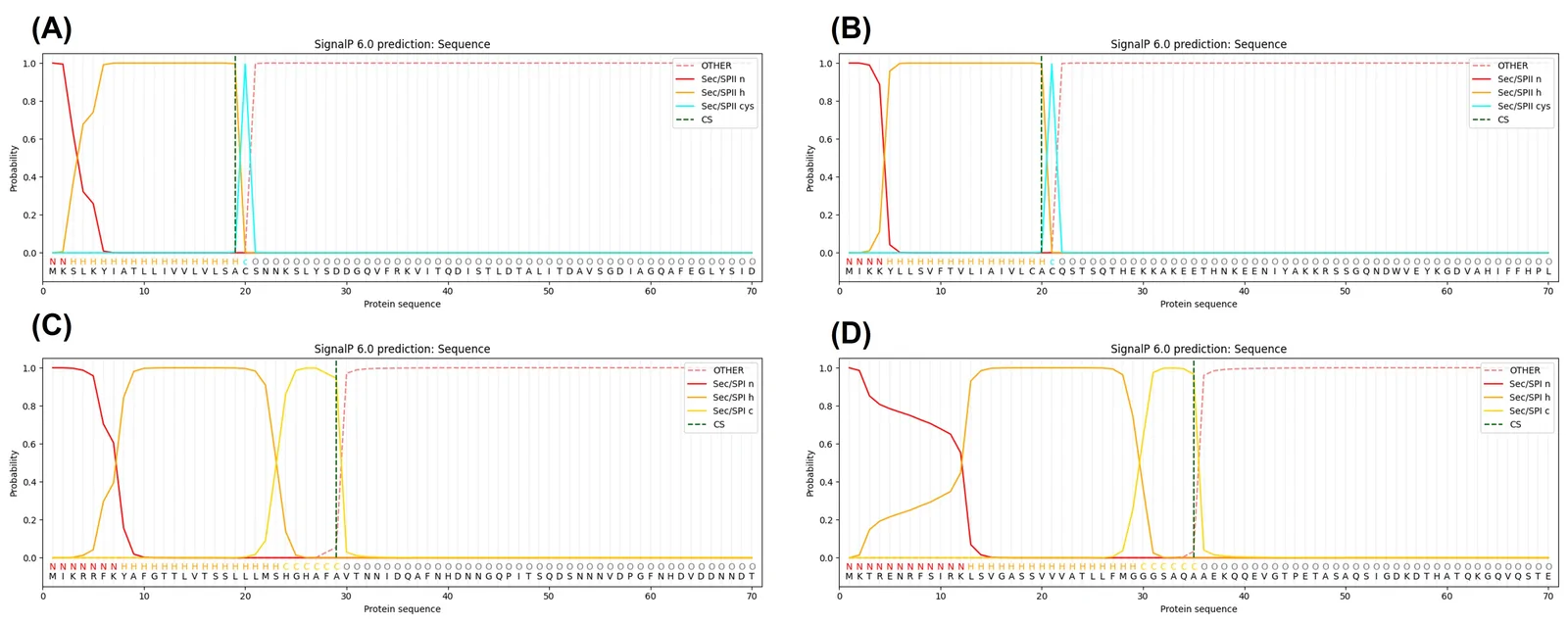
Signal peptide prediction of the selected surface proteins using SignalP 6.0. (**A**) Peptide ABC transporter substrate-binding protein. (**B**) Polysaccharide deacetylase. (**C**) LPXTG cell wall anchor domain-containing protein. (**D**) YSIRK-type signal peptide-containing protein.

**Figure 3:**
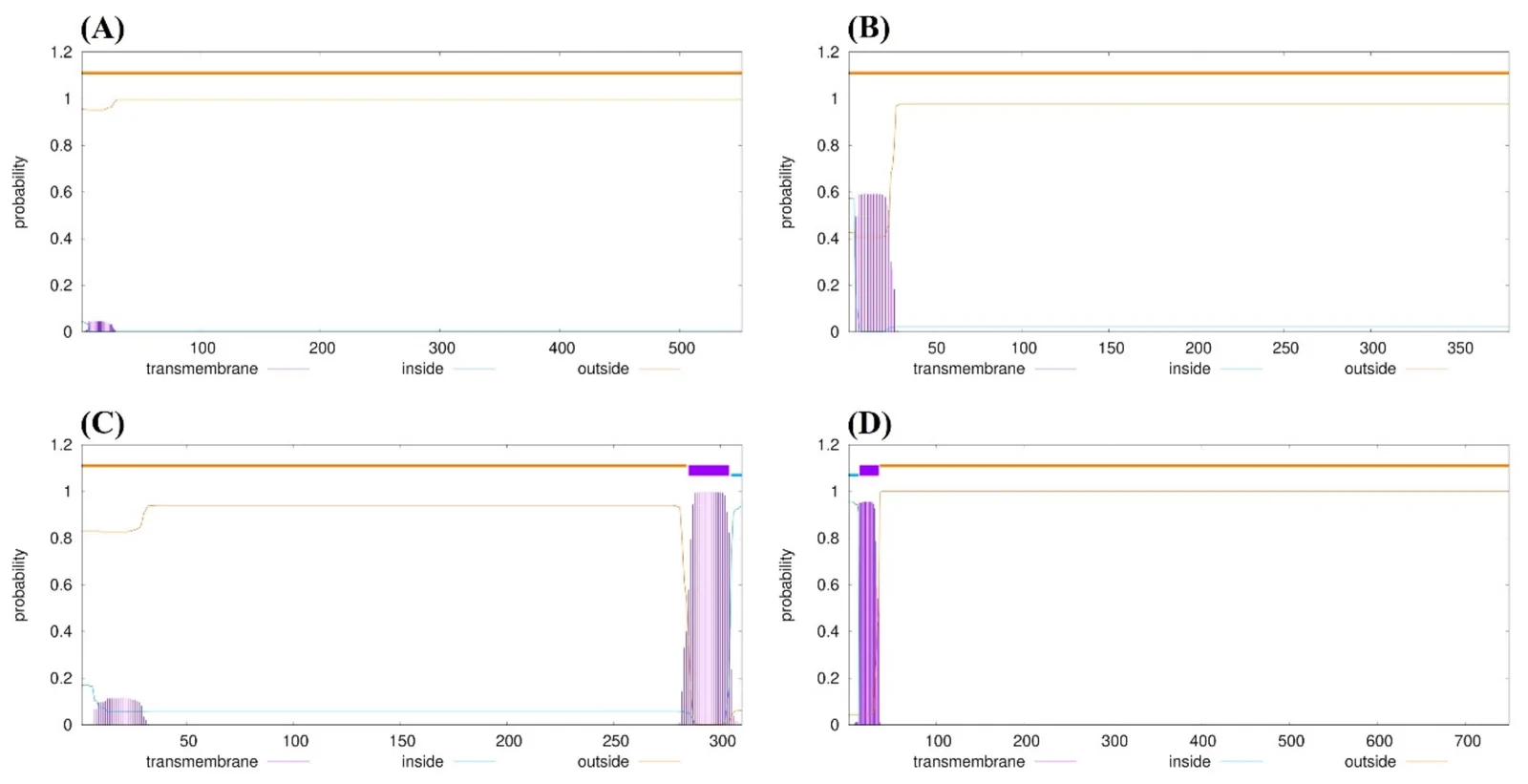
Transmembrane topology prediction of the selected surface proteins using TMHMM. (**A**) Peptide ABC transporter substrate-binding protein. (**B**) Polysaccharide deacetylase. (**C**) LPXTG cell wall anchor domain-containing protein. (**D**) YSIRK-type signal peptide-containing protein.

**Figure 4:**
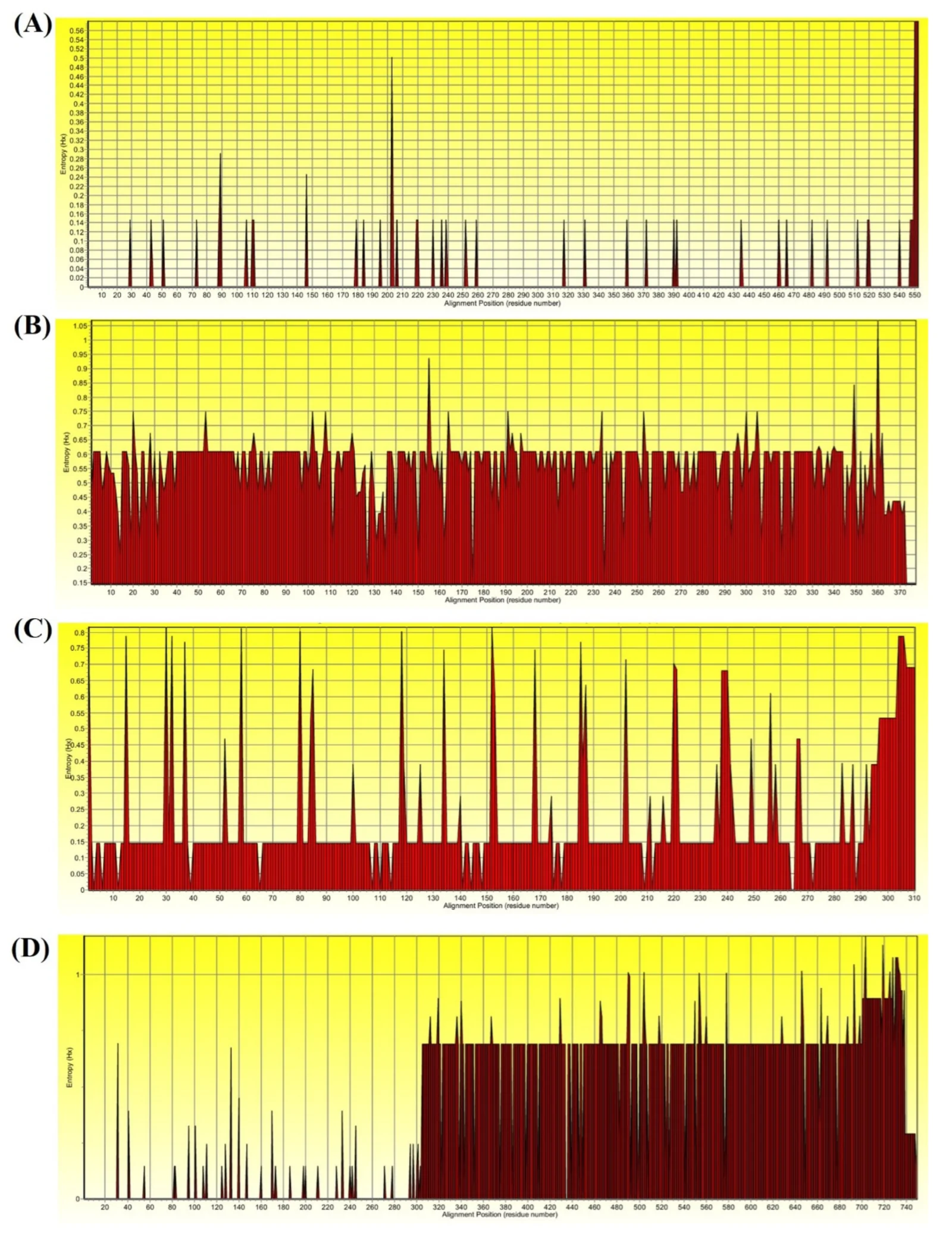
Shannon entropy plots of the selected surface proteins. Red areas/lines indicate the Shannon entropy values, whereas the yellow background is used only for graphical visualization. (**A**) Peptide ABC transporter substrate-binding protein. (**B**) Polysaccharide deacetylase. (**C**) LPXTG cell wall anchor domain-containing protein. (**D**) YSIRK-type signal peptide-containing protein.

**Figure 5:**
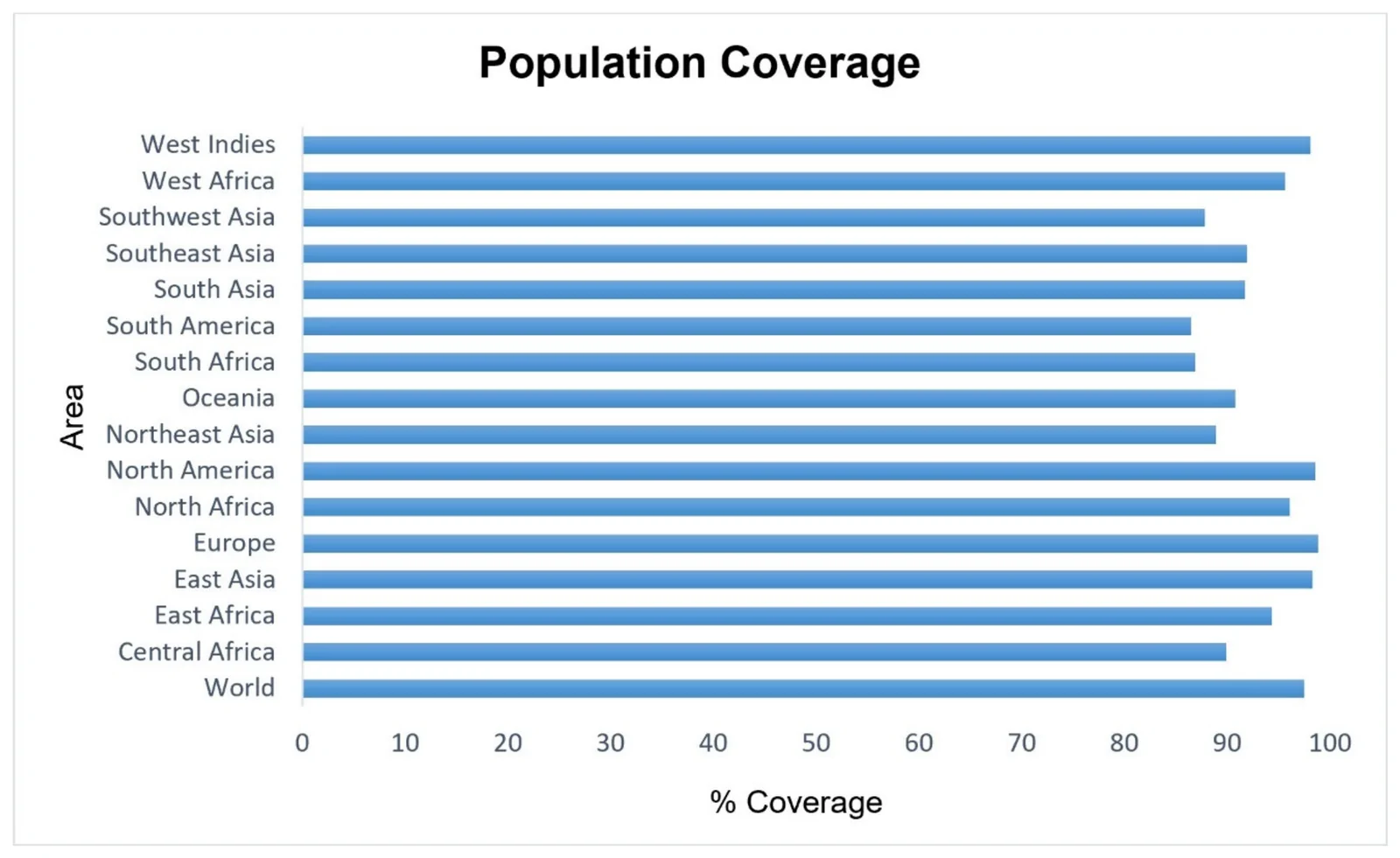
Regional and worldwide population coverage predicted for the selected T-cell epitope set.

**Figure 6:**
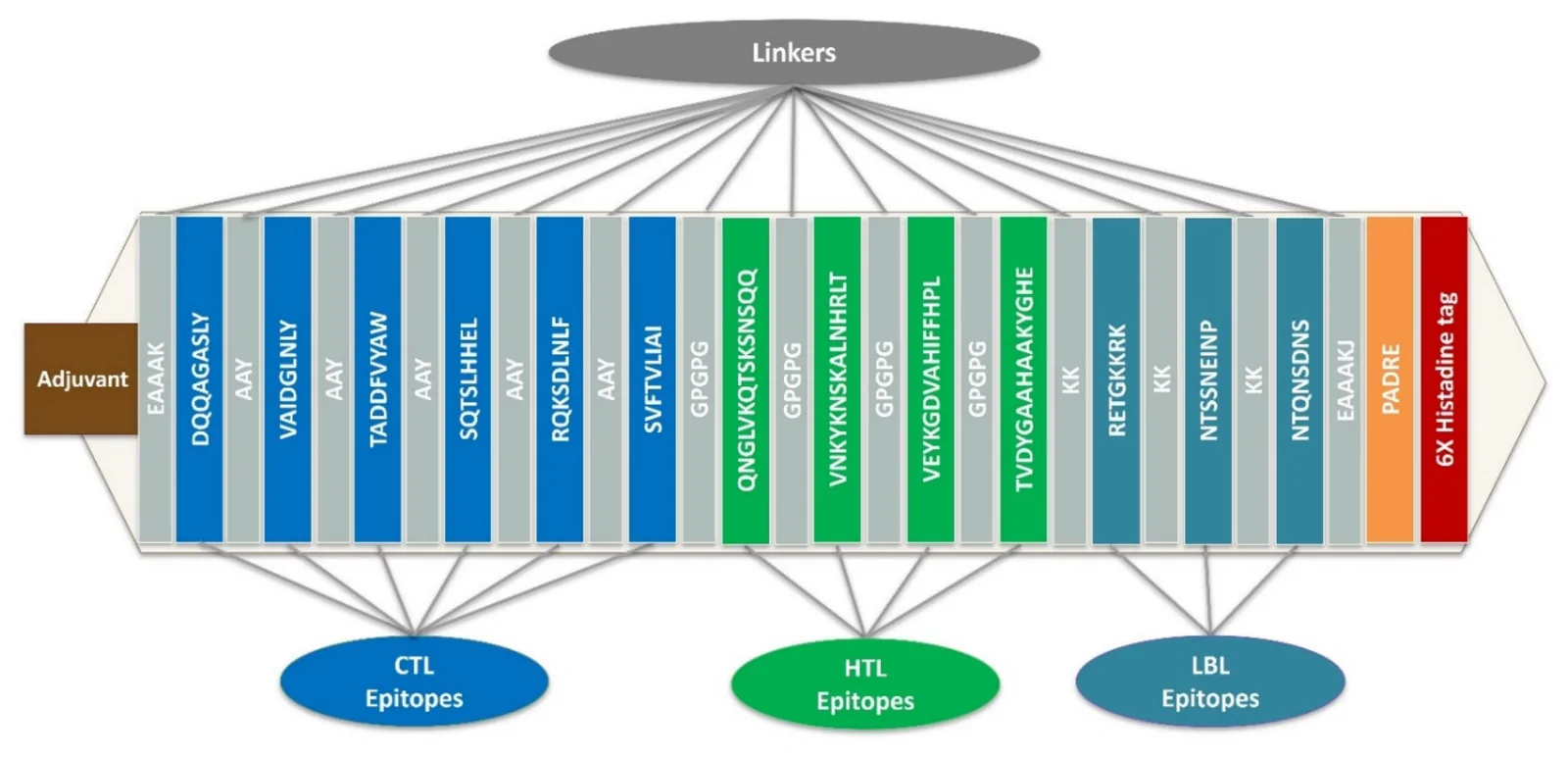
Schematic layout of the final vaccine construct.

**Figure 7:**
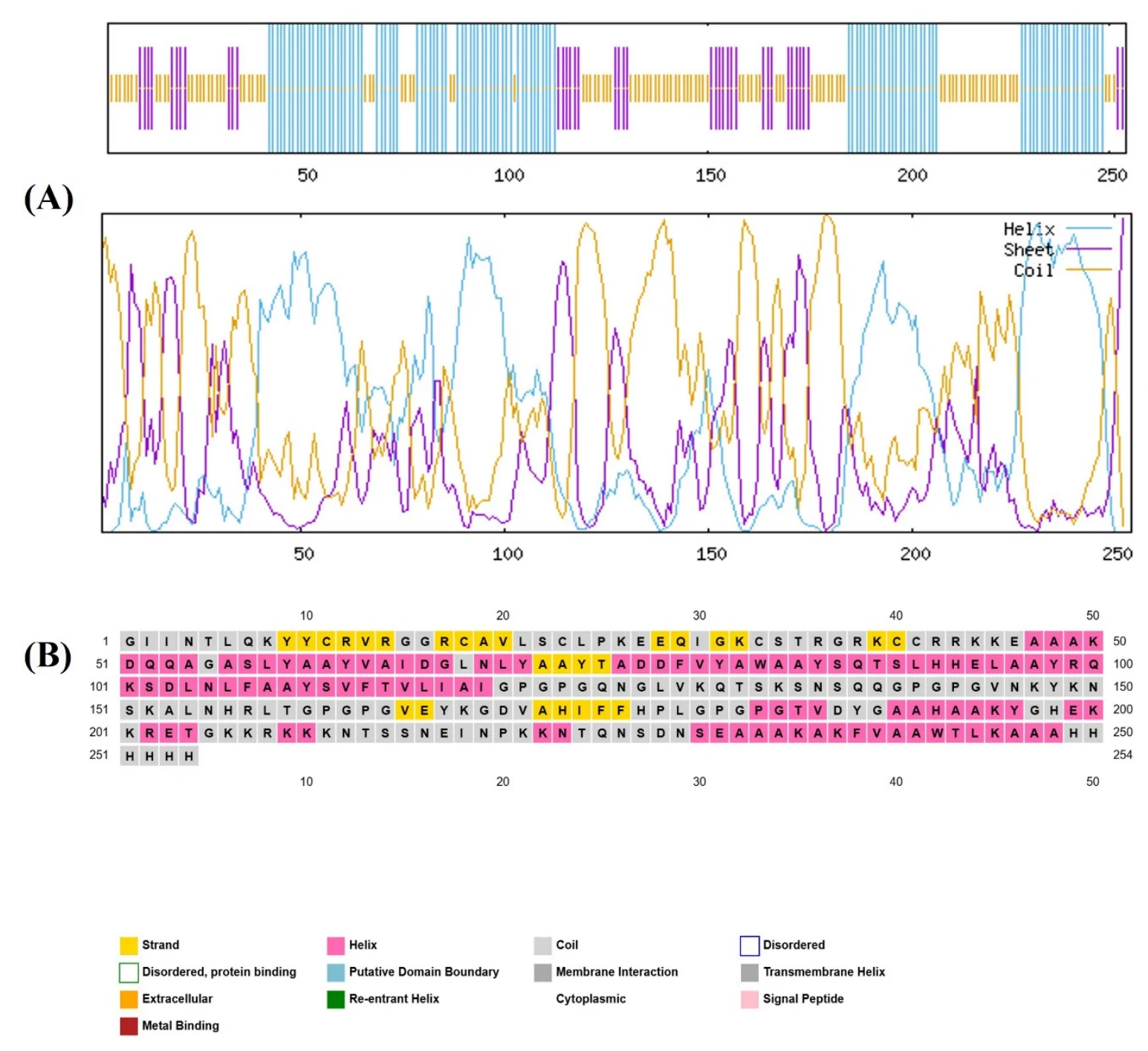
Computational prediction of the secondary structure of the vaccine construct. (**A**) Structural element distribution obtained from GOR4. (**B**) Residue-wise structural assignment produced by PSIPRED.

**Figure 8:**
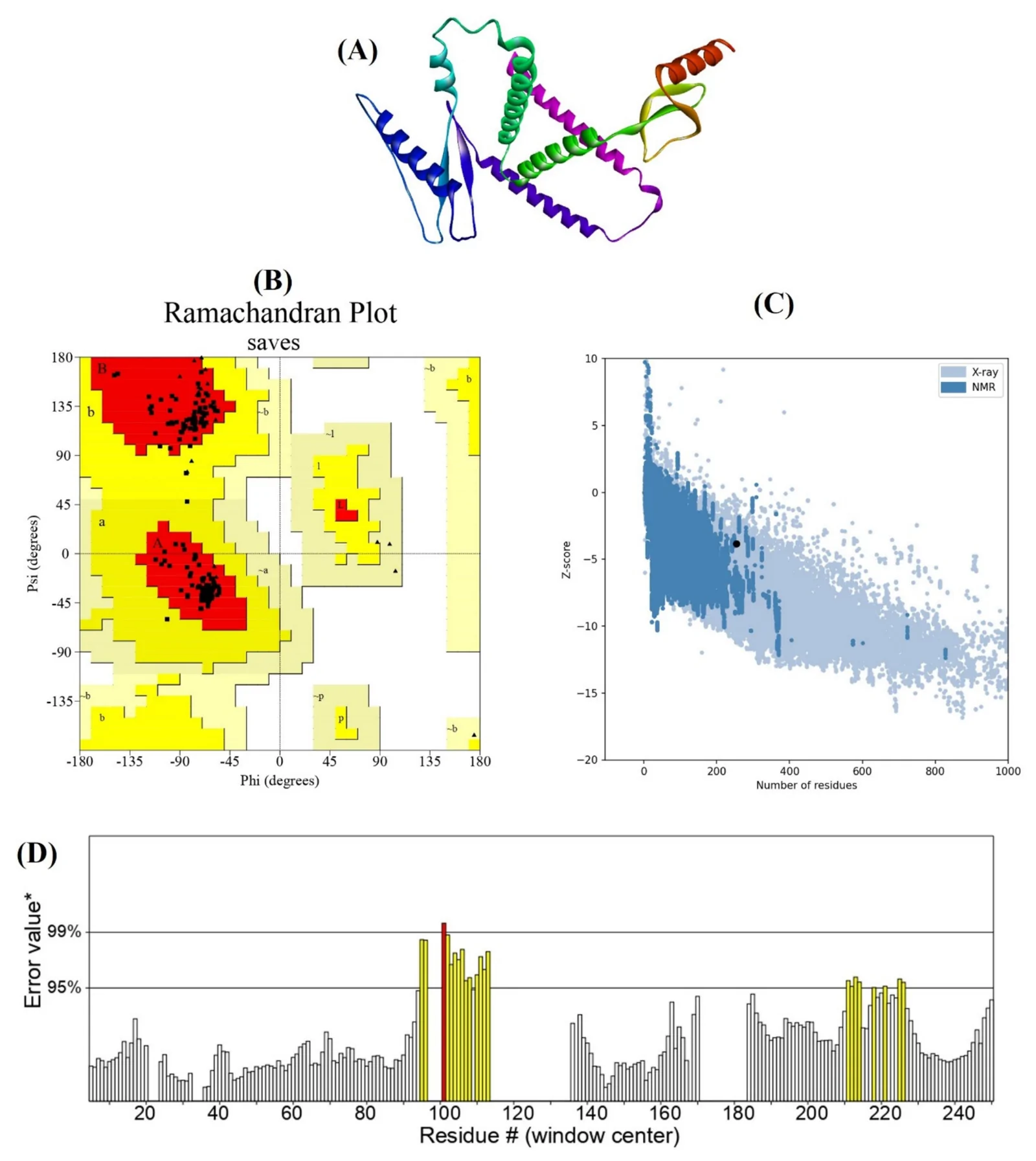
Structural evaluation of the vaccine construct. (**A**) Three-dimensional model generated by AlphaFold3. (**B**) PROCHECK-derived Ramachandran plot summarizing residue conformations, the red regions indicate the most favored regions, the yellow regions indicate additionally allowed regions, the light-yellow regions indicate generously allowed regions, and the white regions indicate disallowed regions. The black squares represent the residues plotted according to their backbone dihedral angles, while the letters indicate the corresponding conformational regions. (**C**) ProSA-web quality assessment based on the calculated Z-score, the light-blue and dark-blue dots represent the X-ray and NMR reference datasets, respectively, as indicated in the figure legend, and the black dot indicates the Z-score of the evaluated structure. (**D**) ERRAT output displaying residue-specific error distribution, yellow bars indicate regions with error values above the 95% confidence level, while the red bar indicates an error value above the 99% confidence level, and the overall quality score of the refined structure.

**Figure 9:**
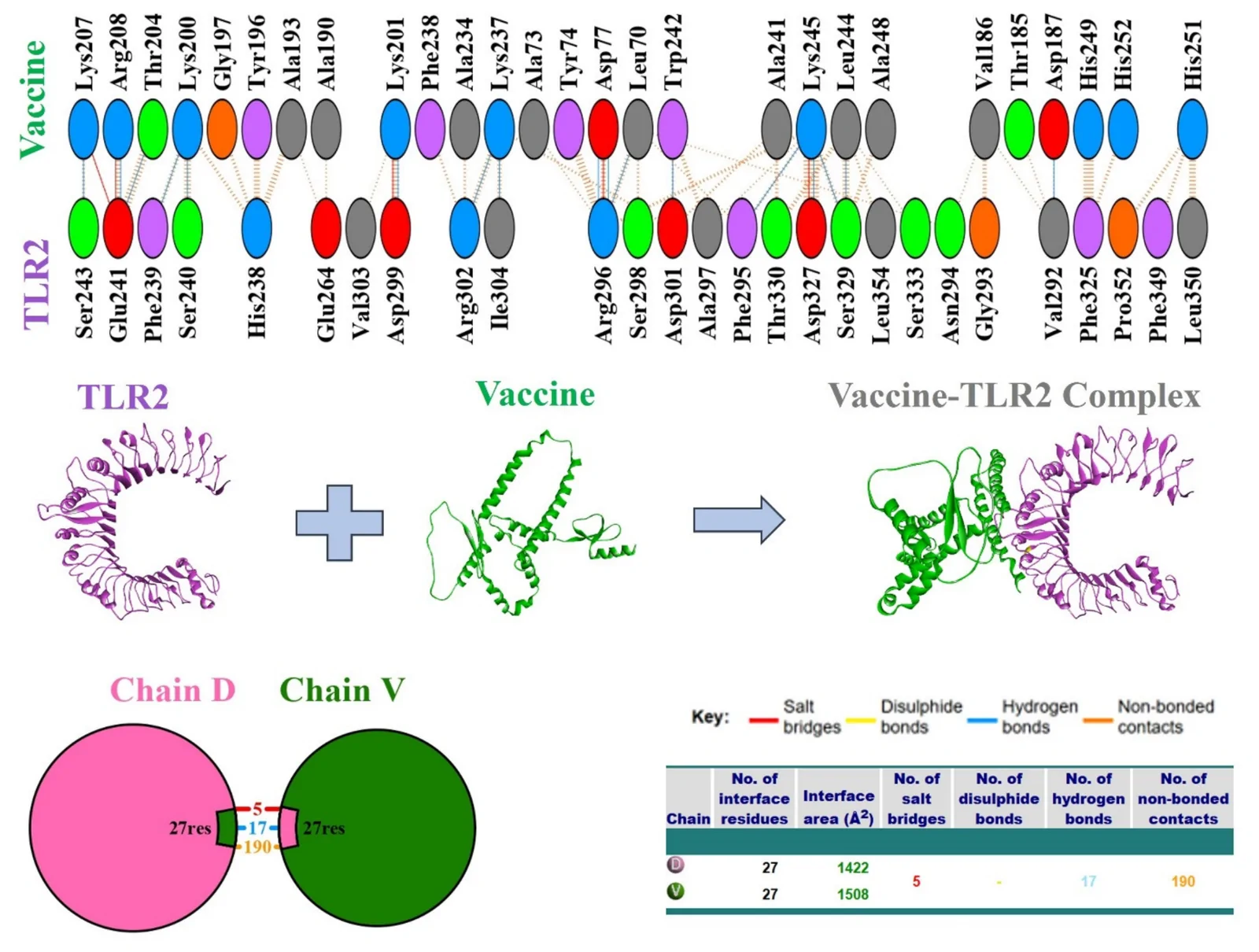
Structural interaction analysis of the vaccine–TLR2 complex.

**Figure 10:**
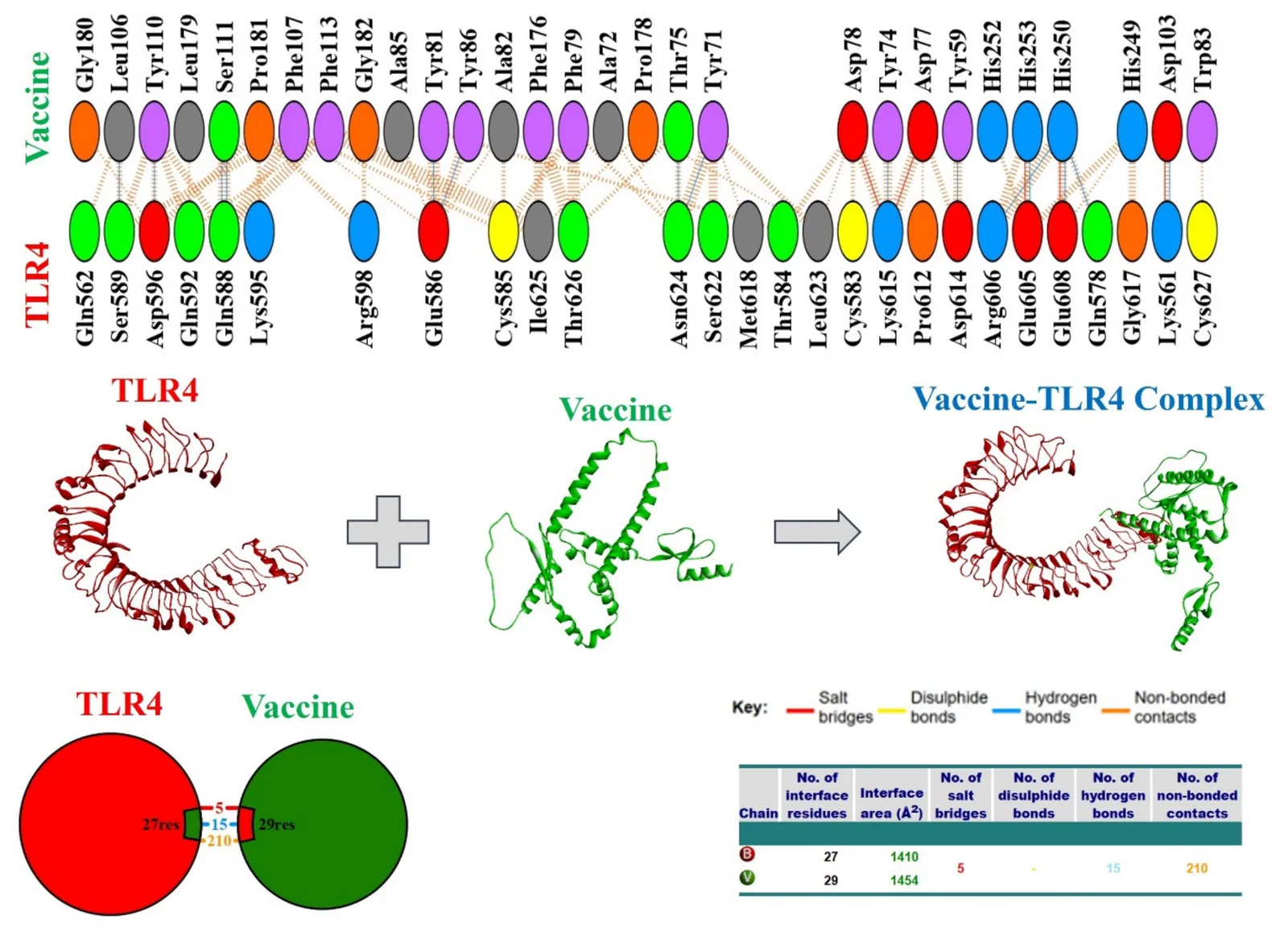
Structural interaction analysis of the vaccine–TLR4 complex.

**Figure 11:**
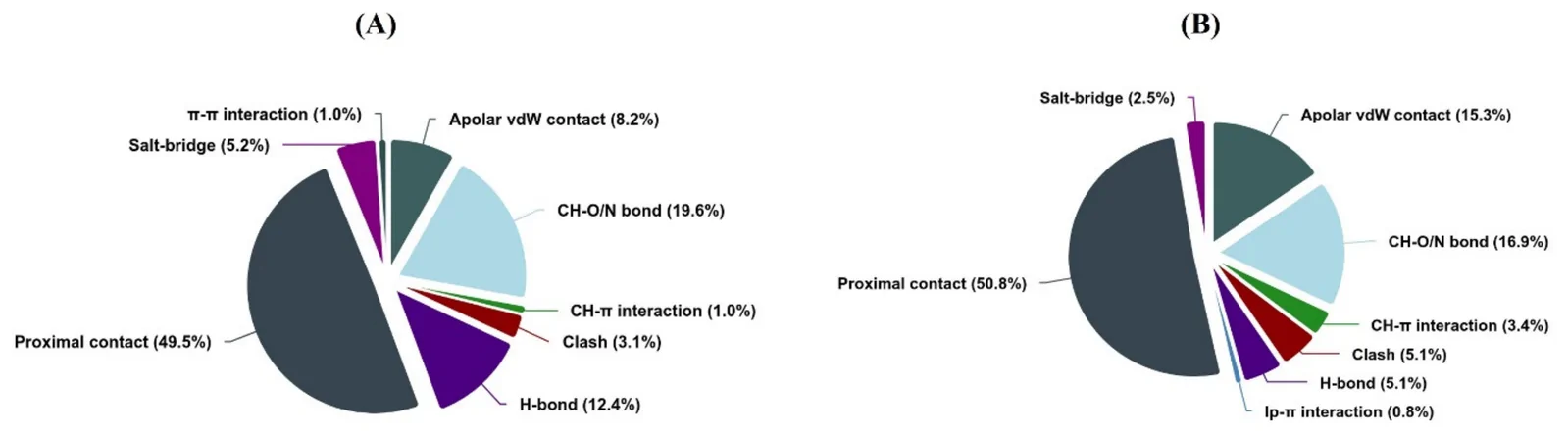
Classification of residue contacts formed following vaccine binding to Toll-like receptors. (**A**) Contacts observed in the TLR2 complex. (**B**) Contacts observed in the TLR4 complex.

**Figure 12:**
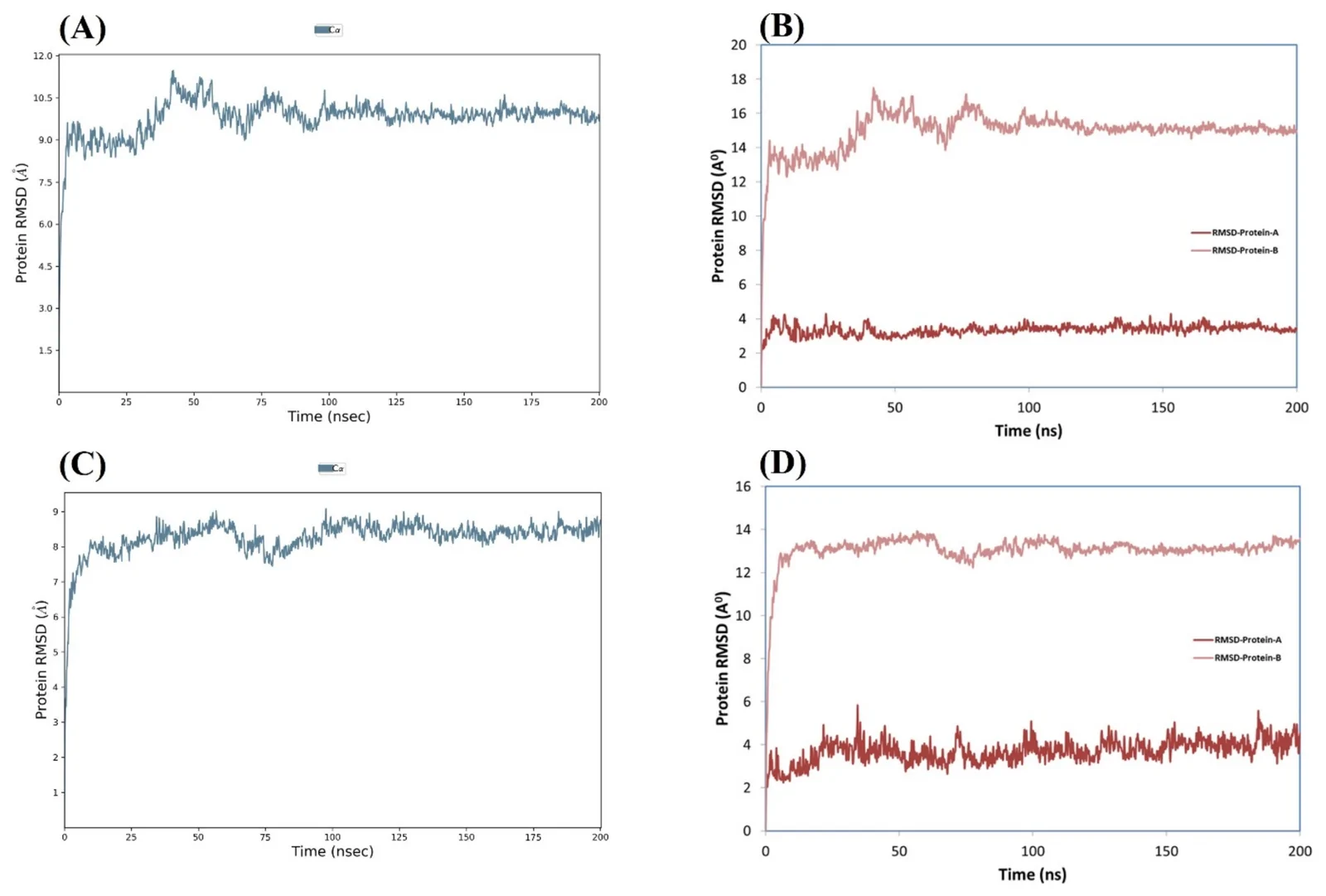
RMSD profiles of the vaccine bound to TLR2 and TLR4 during the 200 ns simulations. (**A**) TLR2–vaccine complex. (**B**) TLR2 (Protein A) and vaccine (Protein B). (**C**) TLR4–vaccine complex. (**D**) TLR4 (Protein A) and vaccine (Protein B).

**Figure 13:**
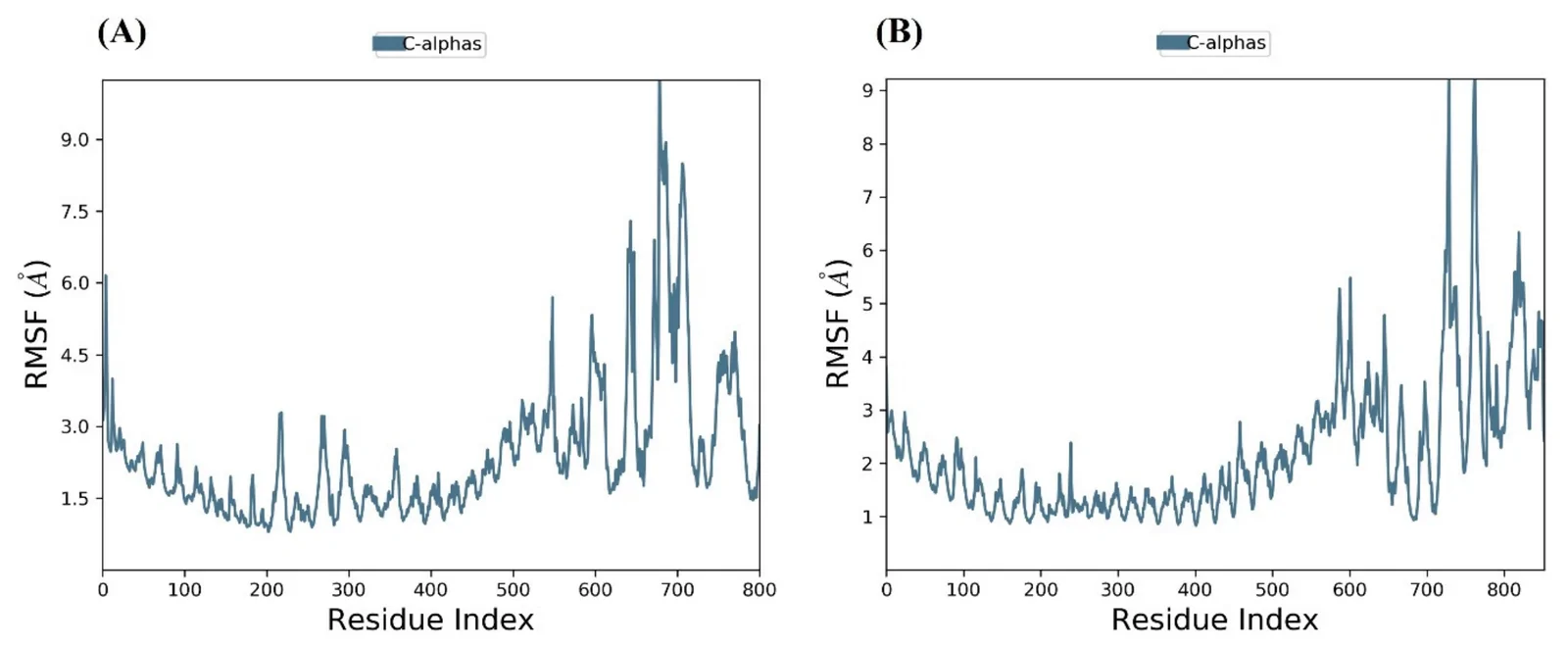
Residue fluctuation profiles obtained from 200 ns MD simulations of the TLR2–vaccine and TLR4–vaccine complexes. (**A**) Residue-wise RMSF of the vaccine–TLR2 complex, where residues 1–549 correspond to TLR2 and residues 550–803 correspond to the vaccine construct. (**B**) Residue-wise RMSF of the vaccine–TLR4 complex, where residues 1–601 correspond to TLR4 and residues 602–855 correspond to the vaccine construct.

**Figure 14:**
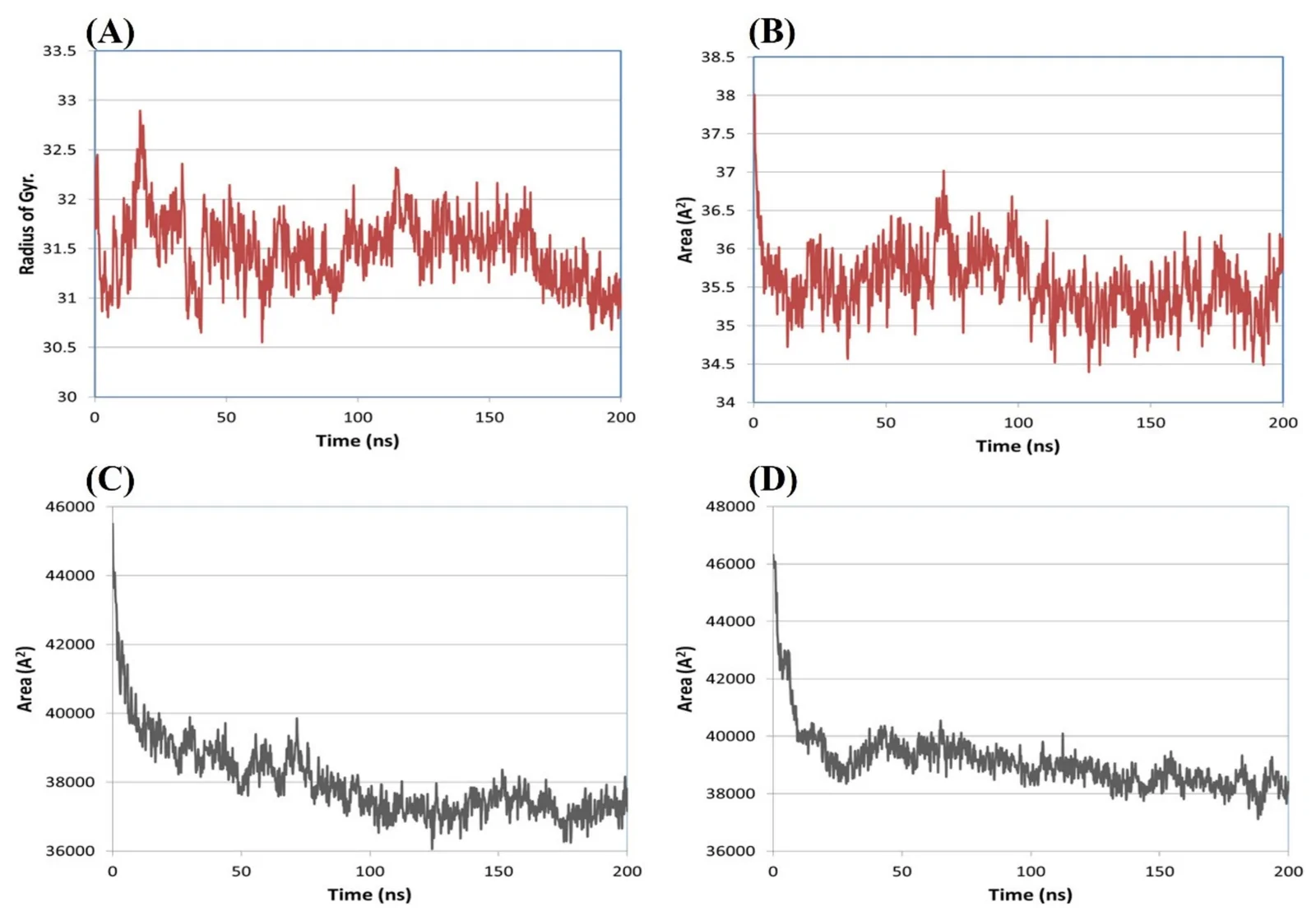
Time-dependent Rg and SASA profiles for the TLR2- and TLR4-bound vaccine systems. (**A**) Rg of the TLR2-bound system. (**B**) Rg of the TLR4-bound system. (**C**) SASA profile of the TLR2-bound system. (**D**) SASA profile of the TLR4-bound system.

**Figure 15:**
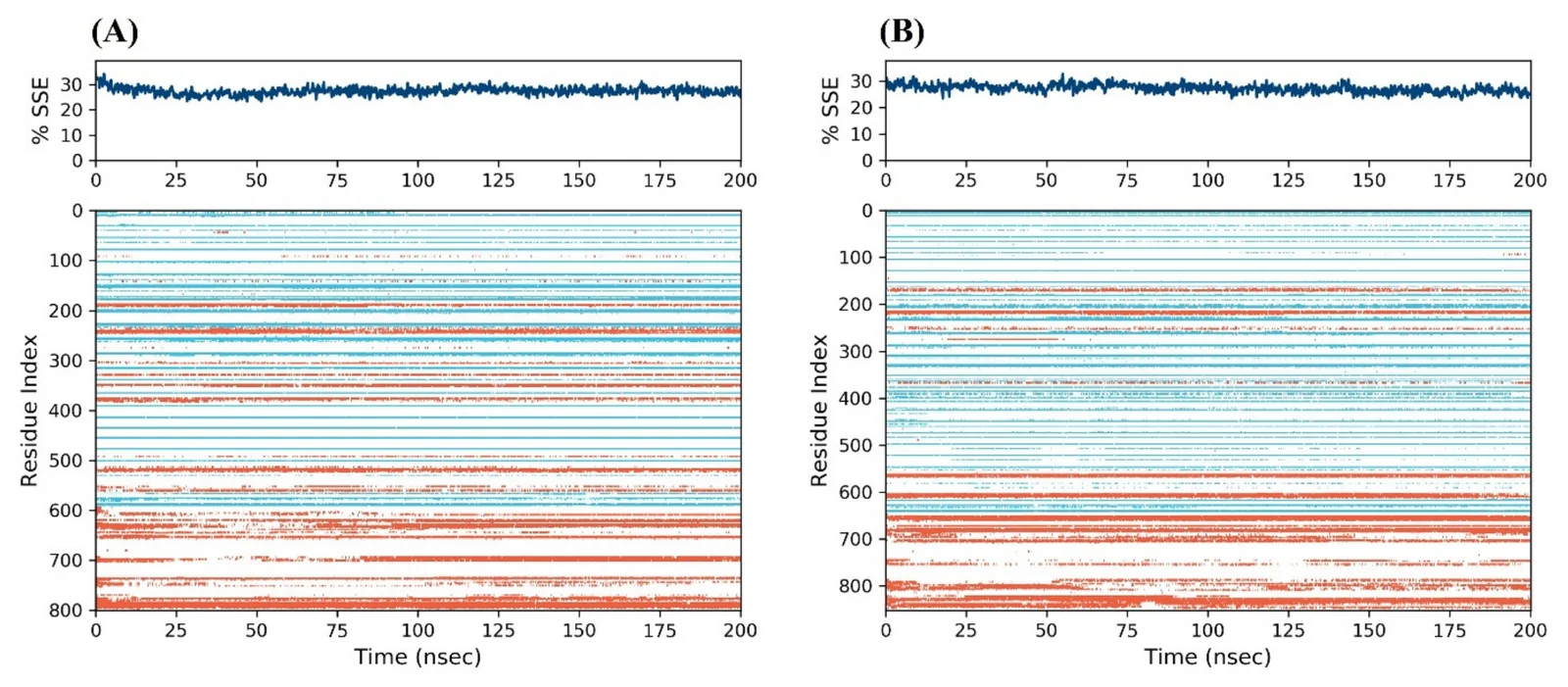
Time-resolved secondary structure profiles for the TLR2- and TLR4-bound vaccine systems over the 200 ns simulations. (**A**) Secondary structure profile of the TLR2-bound system. (**B**) Secondary structure profile of the TLR4-bound system. Cyan regions indicate β-strand structures, whereas orange regions indicate α-helical structures. White regions represent residues not assigned to these secondary-structure elements.

**Figure 16:**
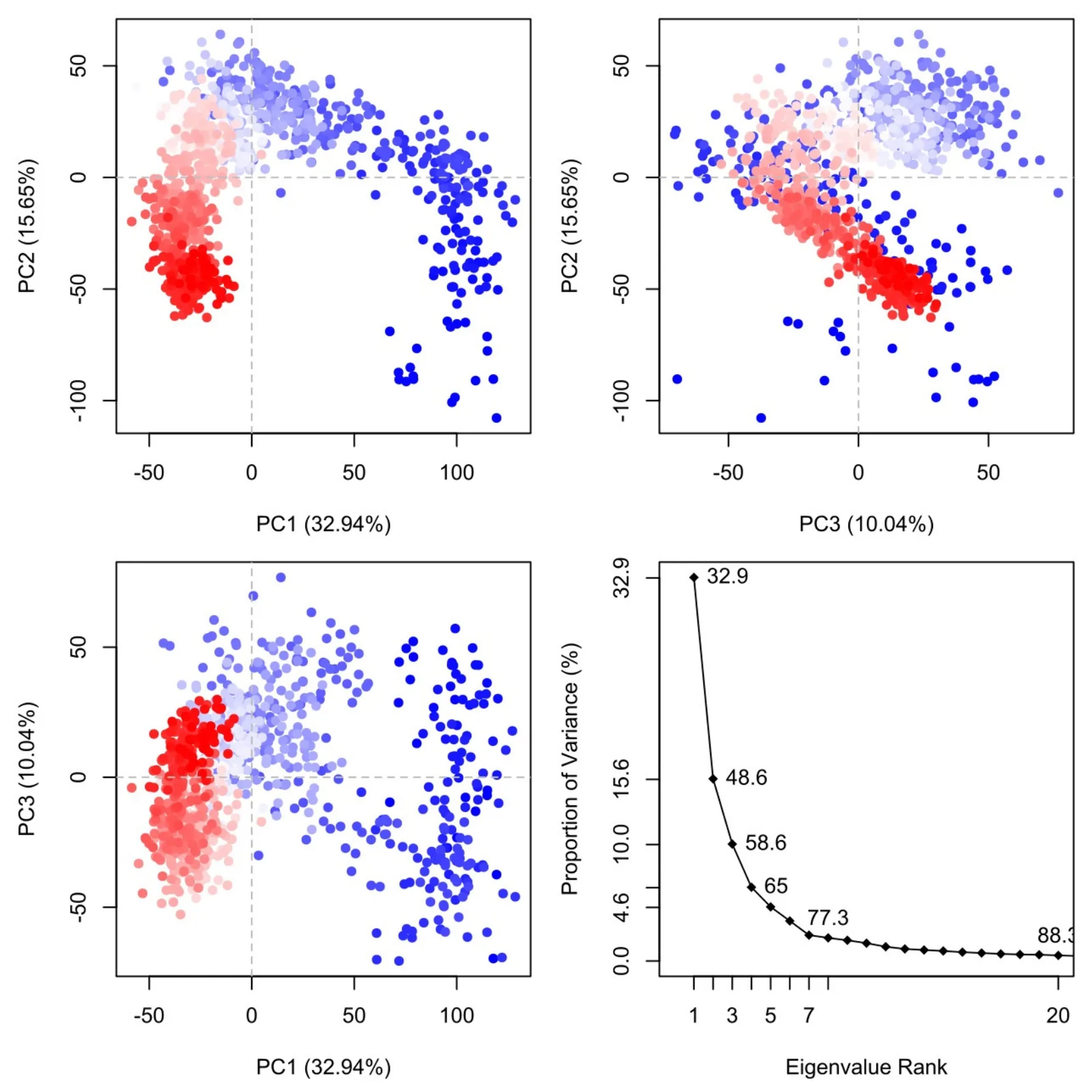
Principal component projections illustrating the conformational space explored by the TLR2-bound vaccine system throughout the 200 ns simulation. Red, light-blue, and dark-blue dots, including intermediate shades, represent the progression of trajectory frames throughout the simulation.

**Figure 17:**
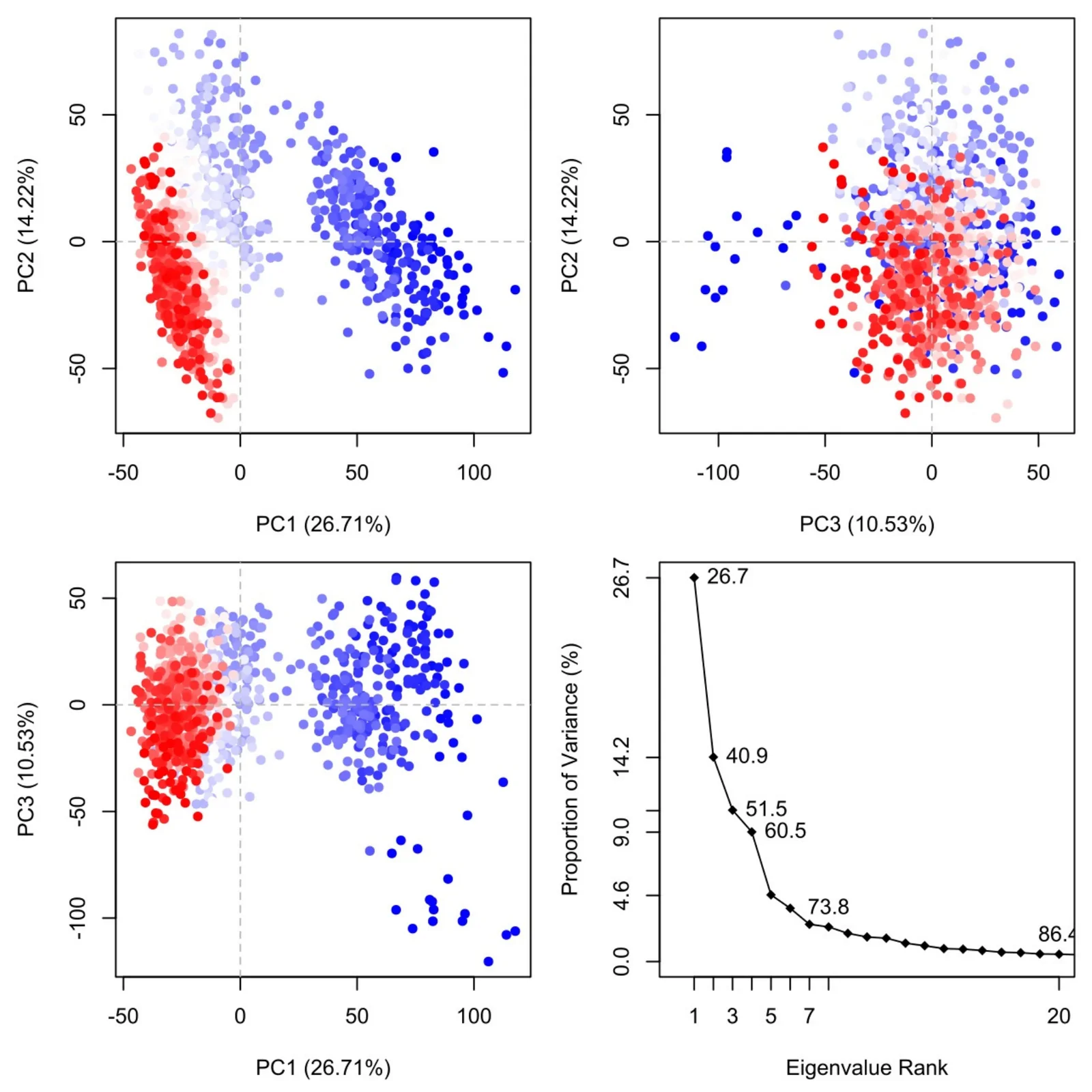
Principal component analysis illustrating the conformational landscape of the TLR4-bound vaccine system. Red, light-blue, and dark-blue dots, including intermediate shades, represent the progression of trajectory frames throughout the simulation.

**Figure 18:**
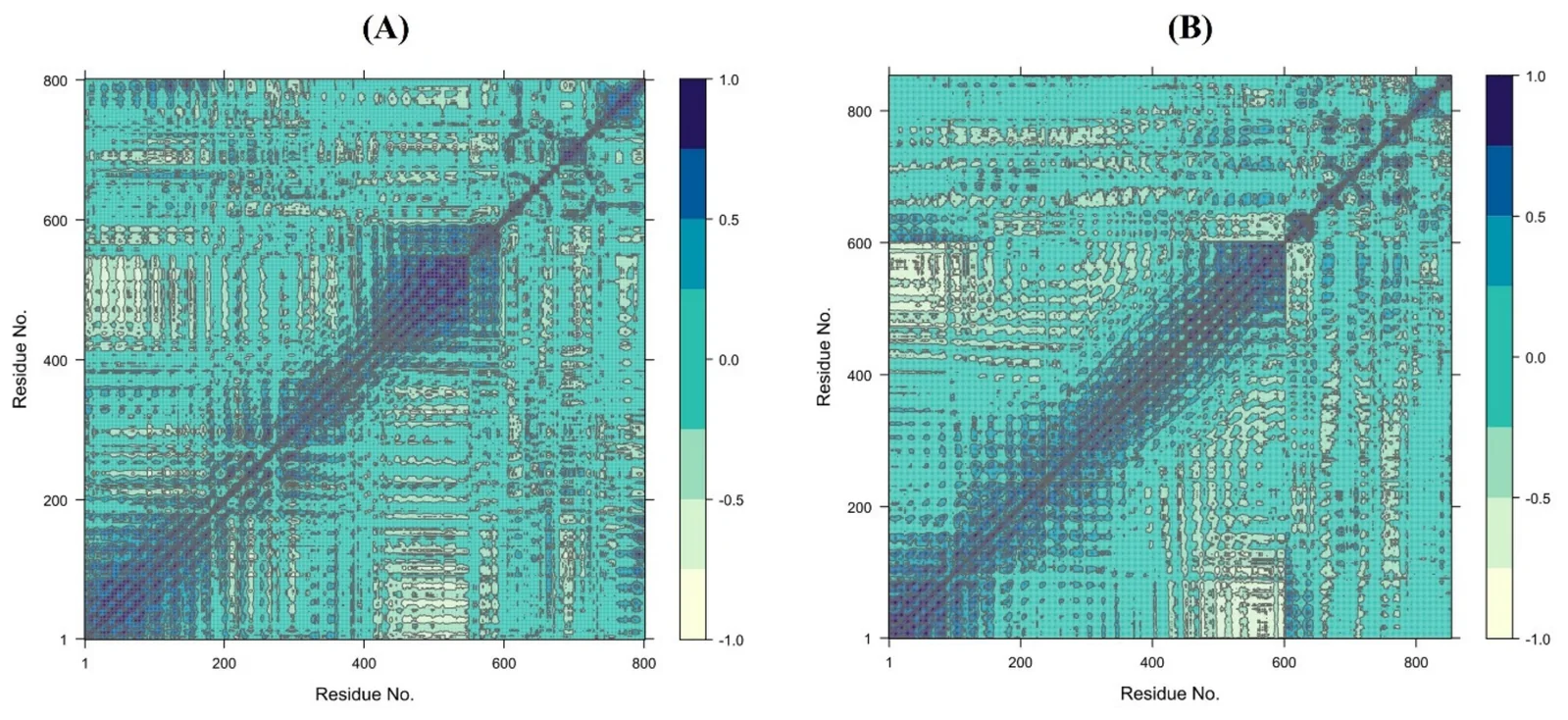
DCCM profiles obtained from the receptor–vaccine systems across the 200 ns MD trajectories. (**A**) Correlation map for the vaccine–TLR2 system. (**B**) Correlation map for the vaccine–TLR4 system.

**Figure 19:**
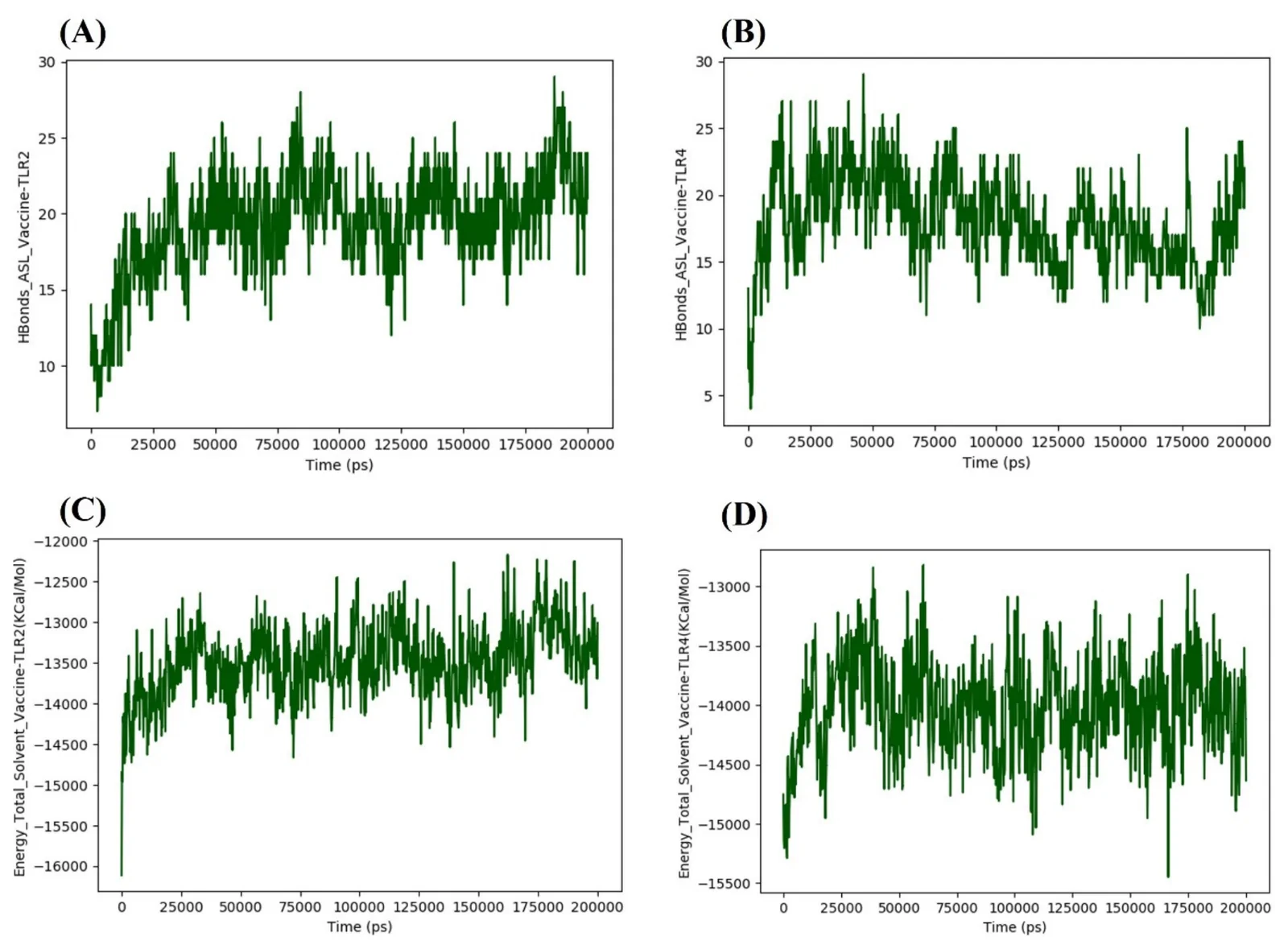
Time-dependent profiles of hydrogen-bond formation and interaction energy for the receptor–vaccine systems across the 200 ns MD trajectories. (**A**) Hydrogen-bond variation in the vaccine–TLR2 system. (**B**) Hydrogen-bond variation in the vaccine–TLR4 system. (**C**) Interaction energy profile of the vaccine–TLR2 system. (**D**) Interaction energy profile of the vaccine–TLR4 system.

**Figure 20:**
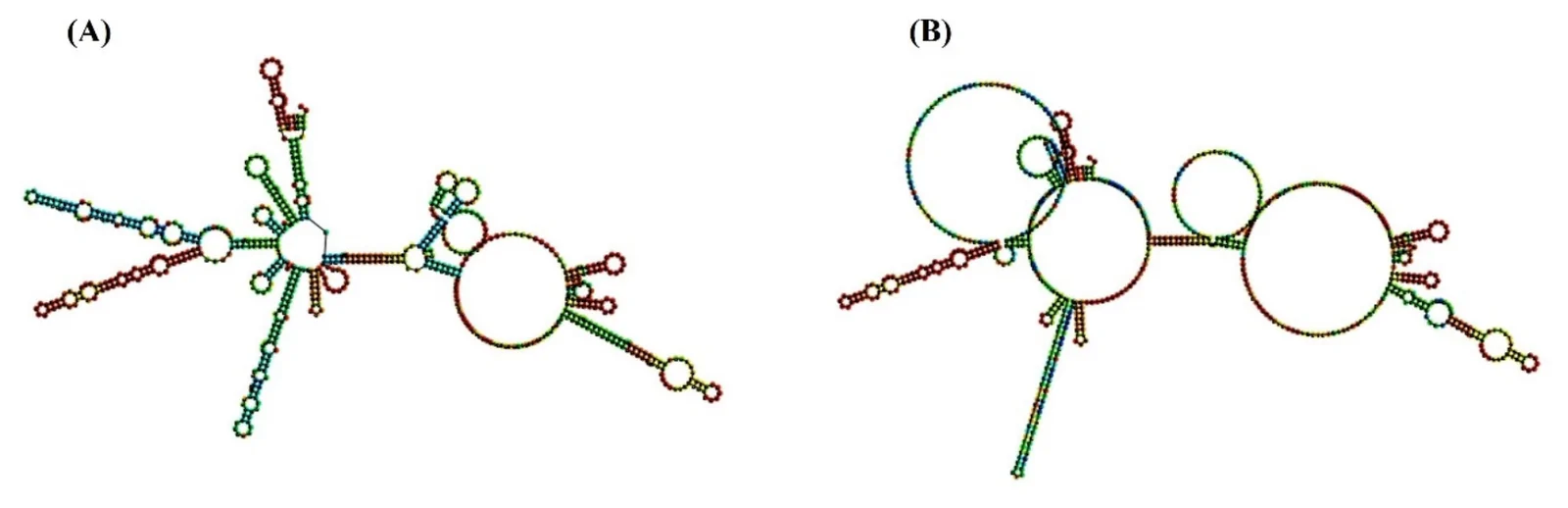
RNAfold-derived structural models of the optimized vaccine mRNA. (**A**) Minimum free-energy (MFE) conformation of the optimized transcript. (**B**) Centroid RNA conformation representing the thermodynamic ensemble. Green indicates stems/canonical helices, red indicates multiloops/junctions, yellow indicates interior loops, blue indicates hairpin loops, and orange indicates 5′ and 3′ unpaired regions.

**Figure 21:**
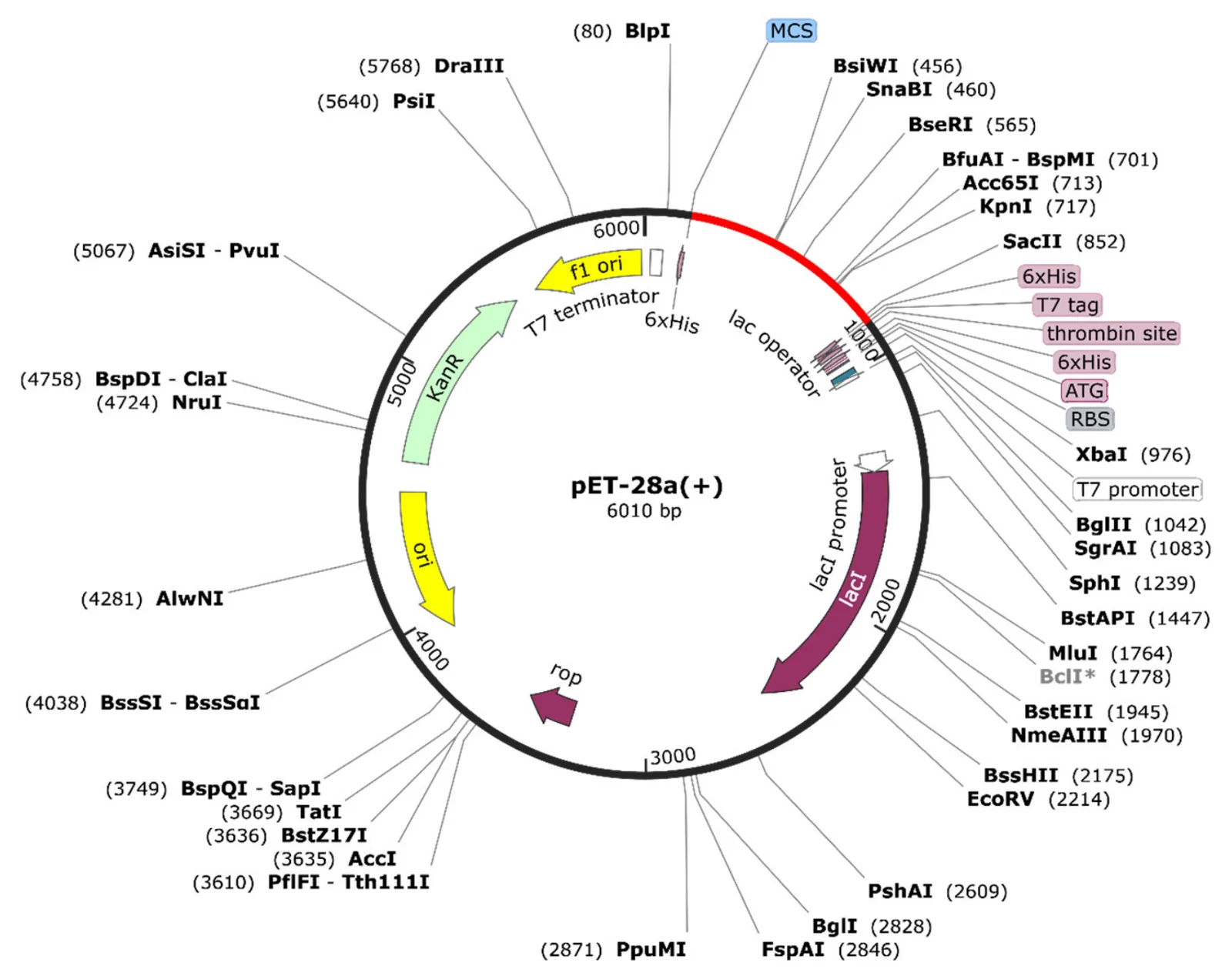
Schematic representation of the recombinant pET-28a(+) plasmid harboring the optimized vaccine coding region following in silico cloning.

**Table 1:** Surface protein candidates identified in *S. haemolyticus* and their properties.

Protein	NCBI Accession ID	Antigenicity	Allergenicity	Subcellular Localization	Transmembrane Topology Prediction		
					Outside	Inside	TMhelix
Peptide ABC transporter substrate-binding protein	QCY38924.1	0.5087 (probable antigen)	Non-allergen	Extracellular	1–552	-	-
Polysaccharide deacetylase	QCY37413.1	0.4823 (probable antigen)	Non-allergen	Extracellular	1–379	-	-
LPXTG cell wall anchor domain-containing protein	QCY37432.1	0.5797 (probable antigen)	Non-allergen	Extracellular	1–284	305–310	285–304
YSIRK-type signal peptide-containing protein	QCY37300.1	0.8197 (probable antigen)	Non-allergen	Extracellular	36–749	1–12	13–35

**Table 2:** Characteristics of the finalized B-cell epitopes.

Protein	Epitope	Position		Antigenicity Score	Allergenicity	Toxicity
		Start	End			
Peptide ABC transporter substrate-binding protein	RETGKKRK	543	550	2.1727 (probable antigen)	Non-allergen	Non-toxic
YSIRK-type signal peptide-containing protein	NTSSNEINP	222	230	1.4597 (probable antigen)	Non-allergen	Non-toxic
	NTQNSDNS	298	305	1.4874 (probable antigen)	Non-allergen	Non-toxic

**Table 3:** Characteristics of the finalized CTL epitopes.

Proteins	Epitope	Position		Antigenicity	Allergenicity	Toxicity	Alleles
		Start	End				
Peptide ABC transporter substrate-binding protein	DQQAGASLY	248	256	1.1271 (probable antigen)	Non-allergen	Non-toxic	HLA-A*26:01, HLA-A*01:01, HLA-B*15:01, HLA-A*30:02, HLA-B*35:01, HLA-B*44:03, HLA-B*44:02
	TADDFVYAW	110	118	0.8601 (probable antigen)	Non-allergen	Non-toxic	HLA-B*53:01, HLA-B*58:01, HLA-B*57:01, HLA-B*35:01, HLA-A*32:01, HLA-A*01:01, HLA-B*44:02, HLA-A*26:01, HLA-B*44:03, HLA-B*51:01
Polysaccharide deacetylase	VAIDGLNLY	340	348	1.0992 (probable antigen)	Non-allergen	Non-toxic	HLA-B*35:01, HLA-A*30:02, HLA-A*26:01, HLA-B*15:01, HLA-B*53:01, HLA-A*01:01, HLA-B*58:01, HLA-B*57:01
	SVFTVLIAI	8	16	0.5645 (probable antigen)	Non-allergen	Non-toxic	HLA-A*68:02, HLA-A*02:06, HLA-A*32:01, HLA-A*02:03, HLA-A*02:01
LPXTG cell wall anchor domain-containing protein	SQTSLHHEL	268	276	0.6085 (probable antigen)	Non-allergen	Non-toxic	HLA-A*02:06, HLA-B*15:01, HLA-B*40:01, HLA-A*32:01, HLA-B*44:02, HLA-B*44:03, HLA-B*08:01, HLA-A*02:01, HLA-A*02:03
YSIRK-type signal peptide-containing protein	RQKSDLNLF	641	649	0.5510 (probable antigen)	Non-allergen	Non-toxic	HLA-B*15:01, HLA-A*32:01, HLA-A*30:02, HLA-A*23:01, HLA-A*24:02, HLA-B*44:02, HLA-B*44:03

**Table 4:** Characteristics of the finalized HTL epitopes.

Proteins	Epitope	Position		Antigenicity	Allergenicity	Toxicity	Alleles
		Start	End				
Peptide ABC transporter substrate-binding protein	VNKYKNSKALNHRLT	271	285	0.8897 (probable antigen)	Non-allergen	Non-toxic	HLA-DRB1*09:01, HLA-DRB1*07:01, HLA-DRB1*04:01, HLA-DRB1*08:02, HLA-DRB1*11:01, HLA-DRB1*04:05, HLA-DRB1*01:01
Polysaccharide deacetylase	VEYKGDVAHIFFHPL	56	70	0.6825 (probable antigen)	Non-allergen	Non-toxic	HLA-DRB1*03:01, HLA-DRB1*07:01, HLA-DRB1*09:01
LPXTG cell wall anchor domain-containing protein	QNGLVKQTSKSNSQQ	136	150	0.8568 (probable antigen)	Non-allergen	Non-toxic	HLA-DRB1*11:01, HLA-DRB1*08:02, HLA-DRB1*04:01, HLA-DRB1*01:01
YSIRK-type signal peptide-containing protein	TVDYGAAHAAKYGHE	441	455	0.7134 (probable antigen)	Non-allergen	Non-toxic	HLA-DRB1*09:01, HLA-DRB1*07:01, HLA-DRB1*01:01, HLA-DRB1*13:02, HLA-DRB1*04:01, HLA-DRB1*08:02, HLA-DRB1*04:05, HLA-DRB1*12:01, HLA-DRB1*11:01

**Table 5:** Predicted physicochemical characteristics of the vaccine construct.

Parameters	Results
Number of amino acids	254
Theoretical pI	9.75
Molecular weight	27,578.17 Da
Instability index	22.72
Aliphatic index	64.33
Grand average of hydropathicity	−0.665
Solubility	0.862

**Table 6:** Quantitative comparison of receptor-binding interface characteristics for the vaccine complexes formed with TLR2 and TLR4, as determined by COCOMAPS.

Category	Parameter	Vaccine–TLR2	Vaccine–TLR4
Residue interactions	Total interacting residues	71	66
	Interacting receptor residues	39	34
	Interacting vaccine residues	32	32
Interface characteristics	Buried surface area (Å^2^)	2930.2	2873.9
	Interface area (Å^2^)	1465.1	1436.95
	Buried surface area (%)	6.41	6.21
	Polar buried surface area (Å^2^)	1029.3	1083.5
	Polar interface area (Å^2^)	514.65	541.75
	Polar interface (%)	35.13	37.70
	Non-polar buried surface area (Å^2^)	1901.0	1790.4
	Non-polar interface area (Å^2^)	950.5	895.2
	Non-polar interface (%)	64.88	62.30
Atomic interactions	Proximal contacts	48	60
	CH–O/N bonds	19	20
	Hydrogen bonds	12	6
	Apolar van der Waals contacts	8	18
	Salt bridges	5	3
	Steric clashes	3	6
	π–π interactions	1	0
	CH–π interactions	1	4
	lp–π interactions	0	1

**Table 7:** MM-GBSA energy decomposition of the vaccine–TLR2 and vaccine–TLR4 complexes.

Energy Component	Vaccine–TLR2 (kcal/mol)	Vaccine–TLR4 (kcal/mol)
Binding free energy (ΔGbind)	−9.04983	−255.343
Coulomb	468.7526	−339.180
Covalent	27.40817	2.810589
Hydrogen bond	−5.34728	−19.7905
Lipophilic	−40.0646	−59.2539
Packing	−1.74784	−4.57011
Self-contact	−0.69318	1.192635
Solv_GB	−348.488	388.2139
Solv_SA	0.00000	0.00000
van der Waals	−108.870	−224.766

## Data Availability

All data generated or analyzed during this study are included in the manuscript and Supplementary Materials. Additional raw data are available from Ahmad Almatroudi upon reasonable request.

## Supplementary Materials

The following supporting information can be downloaded at: https://www.mdpi.com/article/10.3390/microorganisms14102152/s1, Supplementary Material S1: The 41 proteins used for the initial screening. Supplementary Material S2: Results of sequence homology analysis of the selected epitopes against human proteins. Supplementary Material S3: Epitope conservancy analysis results. Supplementary Material S4: Additional raw data, including epitope prediction files, the vaccine three-dimensional model, docking complexes, and molecular dynamics simulation results. The amino acid sequence of the designed vaccine is given below. GIINTLQKYYCRVRGGRCAVLSCLPKEEQIGKCSTRGRKCCRRKKEAAAKDQQAGASLYAAYVAIDGLNLYAAYTADDFVYAWAAYSQTSLHHELAAYRQKSDLNLFAAYSVFTVLIAIGPGPGQNGLVKQTSKSNSQQGPGPGVNKYKNSKALNHRLTGPGPGVEYKGDVAHIFFHPLGPGPGTVDYGAAHAAKYGHEKKRETGKKRKKKNTSSNEINPKKNTQNSDNSEAAAKAKFVAAWTLKAAAHHHHHH.
