# Supplementary material for: In Silico Design and Evaluation of a Multi-Epitope Vaccine Candidate Against Staphylococcus haemolyticus
Source: Microorganisms. 2026 Sep 24;14(10):2152. doi: 10.3390/microorganisms14102152 (PMC13707924; doi:10.3390/microorganisms14102152)
Supplement: Supplementary file 1 [file microorganisms-14-02152-s001.zip › Supplementary Material S2.pdf]

Sequence homology analysis of the selected vaccine epitopes from *Staphylococcus haemolyticus* against *Homo sapiens* reference proteins (taxid: 9606) was performed using BLASTP

Sr.No.	Epitope sequence	Human protein	Matching protein accession	Alignment length with subject (aa)	% Identity	E-value
1	RETGKKRK	platelet-derived growth factor subunit A isoform X1	XP_011513717.1	8	87.50%	1.4
2	NTSSNEINP	SR-related and CTD-associated factor 8 isoform a	NP_001273117.1	7	100%	1.7
3	NTQNSDNS	ankyrin repeat and SOCS box protein 2 isoform 1	NP_001189358.1	7	85.71%	46
4	DQQAGASLY	E3 ubiquitin-protein ligase RNF213 isoform X8	XP_054172769.1	7	85.71%	40
5	TADDFVYAW	CCR4-NOT transcription complex subunit 1 isoform a	NP_057368.3	5	100%	14
6	VAIDGLNLY	N-acetylgalactosamine-6-sulfatase isoform X1	XP_054235973.1	7	100%	2.4
7	SVFTVLIAI	protein SAAL1	NP_612430.2	9	77.78%	28
8	SQTSLHHEL	nesprin-2 isoform X1	XP_011534876.1	6	100%	9.8
9	RQKSDLNLF	protocadherin alpha-C2 isoform 1 precursor	NP_061722.1	8	75%	20
10	VNKYKNSKALNHRLT	zinc finger MYM-type protein 6	NP_009098.3	11	63.64%	5.4
11	VEYKGDVAHIFFHPL	phytanoyl-CoA dioxygenase, peroxisomal isoform b	NP_001032626.1	12	66.67%	5.4
12	QNGLVKQTSKSNSQQ	14-3-3 protein beta/alpha	NP_003395	10	80%	11
13	TVDYGAAHAAKYGHE	zinc finger protein 638 isoform 2	NP_001239542.1	12	66.67%	2.7

Note: Percentage identity represents the proportion of identical amino acid residues within the aligned region between the query epitope and the corresponding human reference protein. Since the selected epitopes are short peptides, the alignment may include only some part of the complete epitope sequence. Therefore, a 100% identity value indicates that all amino acid residues within the aligned region were

identical, but it does not necessarily mean that the entire epitope sequence was identical to the human protein. Percentage identity should therefore be interpreted together with the alignment length and E-value when assessing potential sequence homology with human proteins.
