# Supplementary material for: In Silico Design and Evaluation of a Multi-Epitope Vaccine Candidate Against Staphylococcus haemolyticus
Source: Microorganisms. 2026 Sep 24;14(10):2152. doi: 10.3390/microorganisms14102152 (PMC13707924; doi:10.3390/microorganisms14102152)
Supplement: Supplementary file 1 [file microorganisms-14-02152-s001.zip › Supplementary Material S3.pdf]

Supplementary Table S1. Conservancy assessment of the shortlisted B-cell, CTL, and HTL epitopes across homologous sequences of the Peptide ABC transporter substrate-binding protein.

Sr. No.	Epitopes<br>Acession no.	VNKYKNSKALNHR LT	DQQAGASL Y	TADDFVYAW	RETGKKR K
1	WP_033079765.1	100%	100%	100%	100%
2	WP_011276233.1	100%	100%	100%	100%
3	WP_193625154.1	100%	100%	100%	100%
4	WP_016931181.1	100%	100%	100%	100%
5	PPK17968.1	100%	100%	100%	87.50%
6	MIB6740029.1	100%	100%	100%	100%
7	MCC3664503.1	100%	100%	100%	100%
8	MCE0454534.1	100%	100%	100%	87.50%
9	WP_053035271.1	100%	100%	100%	50%
10	WP_053015825.1	100%	100%	100%	50%
11	WP_308062229.1	100%	100%	100%	50%
12	WP_053018173.1	100%	100%	100%	50%
13	WP_368868540.1	100%	100%	100%	50%
14	WP_049395556.1	100%	100%	100%	50%
15	WP_053038701.1	100%	100%	100%	50%
16	WP_053023769.1	100%	100%	100%	50%
17	WP_368917427.1	100%	100%	100%	50%
18	WP_368871196.1	100%	100%	100%	50%
19	WP_240554726.1	100%	88.89%	100%	50%
20	WP_369078177.1	100%	100%	100%	50%
21	WP_346564494.1	100%	100%	100%	50%

22	WP_308892090.1	100%	100%	100%	50%
23	WP_053037856.1	100%	100%	100%	50%
24	WP_145437175.1	100%	100%	100%	50%
25	WP_070495488.1	100%	100%	88.89%	50%
26	WP_394291986.1	100%	100%	100%	50%
27	WP_240568938.1	100%	100%	100%	50%
28	WP_053019681.1	100%	100%	100%	50%
29	WP_486578502.1	100%	100%	100%	50%
30	WP_053034857.1	100%	100%	100%	50%

Table S2. Conservancy assessment of shortlisted B-cell, CTL, and HTL epitopes across homologous Polysaccharide deacetylase

**Sr. No.**	**Epitopes**	**VEYKGDVAHI FFHPL**	**VAIDGLNLY**	**SVFTVLIAI**
	**Acession no.**			
1	WP_059747058.1	100%	100%	100%
2	HDN2211894.1	100%	100%	100%
3	MBY6179734.1	100%	88.89%	100%
4	WP_033079782.1	100%	100%	100%
5	MDU7039400.1	100%	100%	100%
6	WP_232142985.1	100%	100%	100%
7	WP_053035151.1	100%	100%	100%
8	WP_462857319.1	100%	100%	88.89%
9	WP_240559322.1	100%	100%	100%
10	WP_053032652.1	100%	100%	100%
11	WP_053039282.1	100%	100%	100%
12	WP_053032313.1	100%	100%	100%

13	WP_037558225.1	100%	100%	100%
14	WP_053020128.1	100%	100%	100%
15	WP_053040760.1	100%	100%	100%
16	WP_323622063.1	100%	100%	100%
17	WP_053030673.1	100%	100%	100%
18	WP_053035452.1	100%	100%	100%
19	WP_200731283.1	100%	100%	100%
20	WP_046464867.1	100%	100%	100%
21	WP_011274631.1	100%	100%	100%
22	PNH22885.1	100%	100%	100%
23	WP_486559794.1	100%	100%	100%
24	WP_426682828.1	100%	100%	100%
25	WP_248498151.1	100%	100%	100%
26	WP_108892023.1	100%	100%	100%
27	WP_237268459.1	100%	100%	100%
28	WP_070601035.1	100%	100%	100%
29	WP_070834751.1	100%	100%	100%
30	WP_486567653.1	100%	88.89%	100%

Table S3. Conservancy assessment of shortlisted B-cell, CTL, and HTL epitopes across homologous LPXTG cell wall anchor domain-containing protein

**Sr. No**	**Epitopes**	**QNGLVKQTSKSNSQQ**	**SQTSLHHEL**
	**Acession no.**		
1	WP_057504702.1	100%	100%
2	WP_053023541.1	100%	100%

3	MGJ5622553.1	100%	100%
4	WP_200734653.1	100%	100%
5	WP_053028338.1	100%	100%
6	WP_261007966.1	100%	100%
7	WP_053035674.1	100%	100%
8	WP_053021824.1	100%	100%
9	WP_070498149.1	100%	100%
10	WP_462857315.1	100%	100%
11	WP_261007422.1	100%	100%
12	WP_119890342.1	100%	100%
13	WP_053015996.1	100%	100%
14	WP_107609934.1	100%	100%
15	MGJ5604082.1	100%	100%
16	WP_136718775.1	100%	100%
17	WP_053027903.1	100%	100%
18	WP_053018571.1	100%	100%
19	MDU5410822.1	100%	100%
20	WP_476714747.1	100%	100%
21	WP_200707936.1	100%	100%
22	WP_200731143.1	100%	100%
23	WP_233050031.1	100%	100%
24	WP_434995242.1	100%	100%
25	WP_168249279.1	100%	100%
26	WP_261006947.1	100%	100%
27	WP_256278083.1	100%	100%
28	WP_325166604.1	100%	100%

29	WP_261007481.1	100%	100%
30	WP_257243753.1	100%	100%

Table S4. Conservancy assessment of shortlisted B-cell, CTL, and HTL epitopes across homologous YSIRK-type signal peptide-containing protein

**Sr. No.**	**Epitopes**	**TVDYGAAHAA KYGHE**	**RQKSDLNLF**	**NTSSNEINP**	**NTQNSDN S**
	**Acession no.**				
1	WP_037558268.1	100%	100%	100%	100%
2	WP_233641216.1	100%	100%	100%	87.50%
3	WP_311557610.1	100%	100%	100%	100%
4	WP_180522261.1	100%	88.89%	100%	100%
5	WP_053016141.1	100%	100%	100%	87.50%
6	WP_059776633.1	100%	100%	100%	87.50%
7	WP_107609741.1	100%	100%	100%	87.50%
8	WP_053026244.1	100%	100%	100%	87.50%
9	WP_037570096.1	100%	100%	100%	75%
10	WP_053020027.1	100%	100%	100%	75%
11	WP_107611986.1	100%	100%	100%	100%
12	WP_107614203.1	100%	100%	100%	87.50%
13	WP_486578539.1	100%	100%	100%	100%
14	WP_462857337.1	100%	100%	100%	100%
15	WP_053024126.1	100%	88.89%	100%	87.50%
16	WP_107637400.1	100%	100%	100%	100%
17	WP_053023179.1	100%	88.89%	100%	87.50%
18	WP_240560635.1	100%	88.89%	100%	87.50%

19	WP_180552026.1	100%	100%	100%	100%
20	WP_085060256.1	100%	100%	88.89%	87.50%
21	WP_285490669.1	100%	100%	100%	100%
22	WP_070663167.1	100%	100%	100%	87.50%
23	WP_053025750.1	100%	88.89%	100%	87.50%
24	WP_193624403.1	100%	100%	100%	100%
25	WP_053032252.1	100%	88.89%	100%	87.50%
26	WP_168249386.1	100%	100%	100%	75%
27	WP_316049003.1	100%	100%	100%	100%
28	WP_136737521.1	100%	88.89%	100%	87.50%
29	WP_316050001.1	100%	100%	100%	100%
30	WP_104948235.1	100%	88.89%	100%	87.50%
