# Supplementary material for: In Silico Design and Evaluation of a Multi-Epitope Vaccine Candidate Against Staphylococcus haemolyticus
Source: Microorganisms. 2026 Sep 24;14(10):2152. doi: 10.3390/microorganisms14102152 (PMC13707924; doi:10.3390/microorganisms14102152)
Supplement: Supplementary file 1 [file microorganisms-14-02152-s001.zip › Supplementary Material S4/Docking with TLR2 and TLR4/CLuspro scores.pdf]

Vaccine-TLR2

Cluster	Members	Representative	Weighted Score
**0**	45	Center	-934.0
		Lowest Energy	-1136.9
**1**	41	Center	-953.7
		Lowest Energy	-1285.3
**2**	34	Center	-1000.5
		Lowest Energy	-1190.8
**3**	30	Center	-926.9
		Lowest Energy	-1161.3
**4**	28	Center	-1053.1
		Lowest Energy	-1141.3
**5**	26	Center	-1026.3
		Lowest Energy	-1082.6
**6**	24	Center	-965.8
		Lowest Energy	-1029.8
**7**	23	Center	-913.2
		Lowest Energy	-1097.2
**8**	23	Center	-1050.8
		Lowest Energy	-1142.6
**9**	23	Center	-921.0
		Lowest Energy	-1153.4
**10**	21	Center	-1060.7
		Lowest Energy	-1229.7
**11**	20	Center	-1067.5
		Lowest Energy	-1067.5
**12**	19	Center	-923.0
		Lowest Energy	-1004.8
**13**	19	Center	-1159.3
		Lowest Energy	-1159.3
**14**	18	Center	-964.8

Cluster	Members	Representative	Weighted Score
		Lowest Energy	-1059.2
**15**	17	Center	-987.3
		Lowest Energy	-1054.9
**16**	17	Center	-1188.5
		Lowest Energy	-1188.5
**17**	15	Center	-925.0
		Lowest Energy	-1094.9
**18**	15	Center	-1001.9
		Lowest Energy	-1021.1
**19**	14	Center	-943.4
		Lowest Energy	-1099.7
**20**	13	Center	-919.2
		Lowest Energy	-1015.3
**21**	13	Center	-997.2
		Lowest Energy	-1069.4
**22**	12	Center	-1092.8
		Lowest Energy	-1092.8
**23**	12	Center	-943.3
		Lowest Energy	-1019.8
**24**	12	Center	-1173.4
		Lowest Energy	-1173.4
**25**	12	Center	-1138.4
		Lowest Energy	-1138.4
**26**	12	Center	-1119.0
		Lowest Energy	-1119.0
**27**	11	Center	-925.3
		Lowest Energy	-1067.8
**28**	11	Center	-1144.5
		Lowest Energy	-1144.5
**29**	11	Center	-1072.2

Cluster	Members	Representative	Weighted Score
		Lowest Energy	-1072.2

Vaccine-TLR4

Cluster	Members	Representative	Weighted Score
**0**	43	Center	-1130.0
		Lowest Energy	-1195.0
**1**	40	Center	-1005.9
		Lowest Energy	-1183.9
**2**	39	Center	-1055.7
		Lowest Energy	-1115.0
**3**	30	Center	-962.7
		Lowest Energy	-1108.1
**4**	29	Center	-984.9
		Lowest Energy	-1077.9
**5**	23	Center	-1009.2
		Lowest Energy	-1210.4
**6**	22	Center	-1068.9
		Lowest Energy	-1092.2
**7**	22	Center	-1127.0
		Lowest Energy	-1197.5
**8**	21	Center	-941.4
		Lowest Energy	-1102.8
**9**	20	Center	-1017.5
		Lowest Energy	-1057.2
**10**	19	Center	-1118.3
		Lowest Energy	-1158.4
**11**	18	Center	-1002.0
		Lowest Energy	-1116.8

Cluster	Members	Representative	Weighted Score
**12**	17	Center	-954.9
		Lowest Energy	-1006.6
**13**	16	Center	-1165.3
		Lowest Energy	-1165.3
**14**	16	Center	-1184.1
		Lowest Energy	-1186.8
**15**	15	Center	-956.7
		Lowest Energy	-1006.9
**16**	14	Center	-1021.4
		Lowest Energy	-1024.8
**17**	14	Center	-951.1
		Lowest Energy	-1121.9
**18**	14	Center	-1016.7
		Lowest Energy	-1138.2
**19**	14	Center	-958.0
		Lowest Energy	-992.6
**20**	12	Center	-1005.3
		Lowest Energy	-1136.6
**21**	12	Center	-1091.1
		Lowest Energy	-1091.1
**22**	11	Center	-965.2
		Lowest Energy	-1028.8
**23**	11	Center	-1079.5
		Lowest Energy	-1079.5
**24**	11	Center	-1049.3
		Lowest Energy	-1049.3
**25**	10	Center	-1069.1
		Lowest Energy	-1069.2
**26**	10	Center	-1109.9
		Lowest Energy	-1109.9

Cluster	Members	Representative	Weighted Score
**27**	10	Center	-1062.0
		Lowest Energy	-1062.0
**28**	10	Center	-1011.1
		Lowest Energy	-1011.1
**29**	10	Center	-999.0
		Lowest Energy	-1017.8
