# Supplementary material for: In Silico Design and Evaluation of a Multi-Epitope Vaccine Candidate Against Staphylococcus haemolyticus
Source: Microorganisms. 2026 Sep 24;14(10):2152. doi: 10.3390/microorganisms14102152 (PMC13707924; doi:10.3390/microorganisms14102152)
Supplement: Supplementary file 1 [file microorganisms-14-02152-s001.zip › Supplementary Material S4/MD Simulations/Vaccine-TLR2-out_pl_1/Vaccine-TLR2-out_pl_1.pdf]

Simulation Interactions Diagram Report

Simulation Details

Jobname: Vaccine-TLR2
Entry title: Full System

CPU #	Job Type	Ensemble	Temp. [K]	Sim. Time [ns]	# Atoms	# Waters	Charge
1	mdsim	NPT	310.0	200.398	117201	34789	0

Protein Information

Tot. Residues	Prot. Chain(s)	Res. in Chain(s)	# Atoms	# Heavy Atoms	Charge
801	'D', 'V'	ict_values([549, 252]	12626	6294	+14

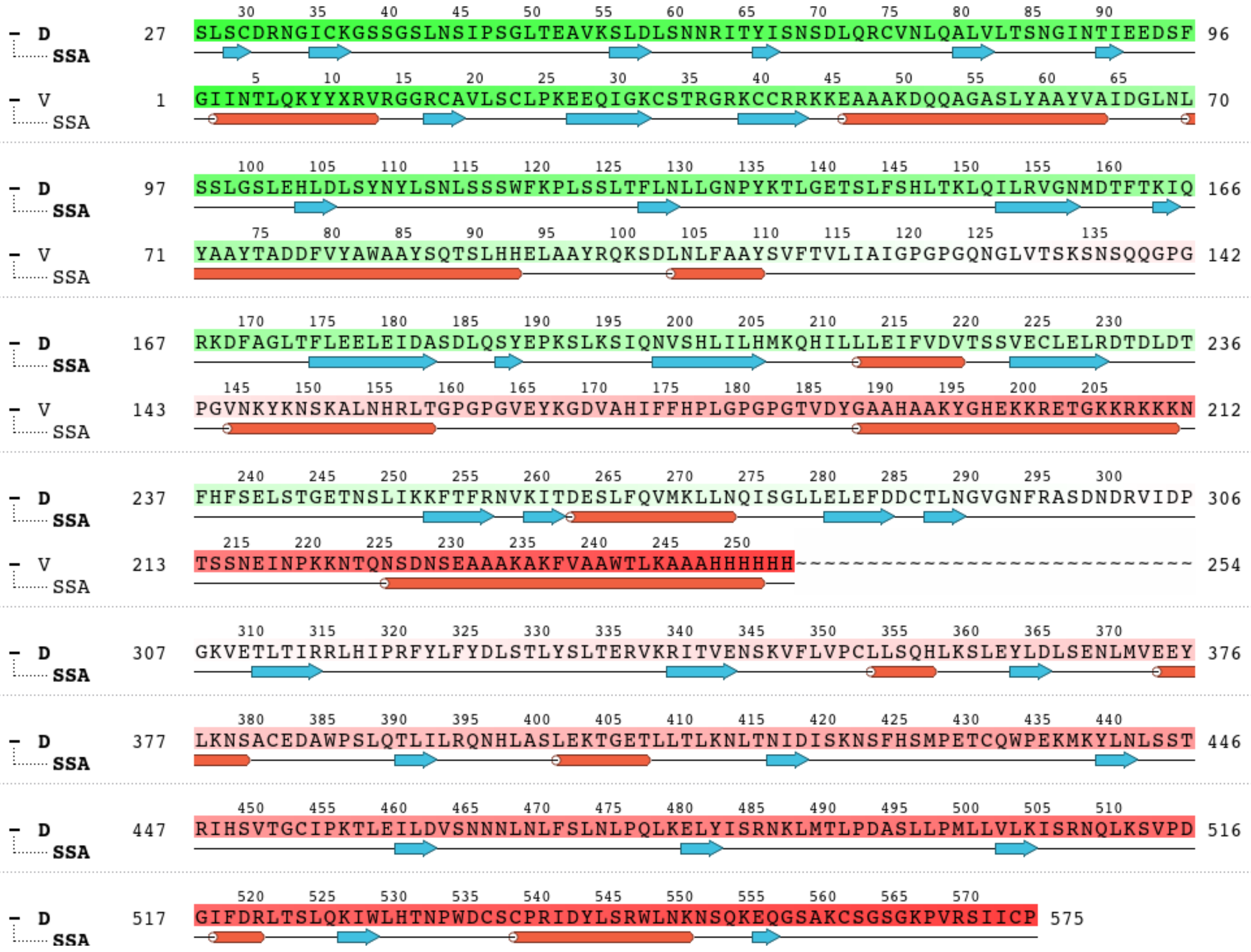

Counter Ion/Salt Information

Type	Num.	Concentration [mM]	Total Charge
Cl	111	58.012	-111
Na	97	50.695	+97

Protein-Ligand RMSD

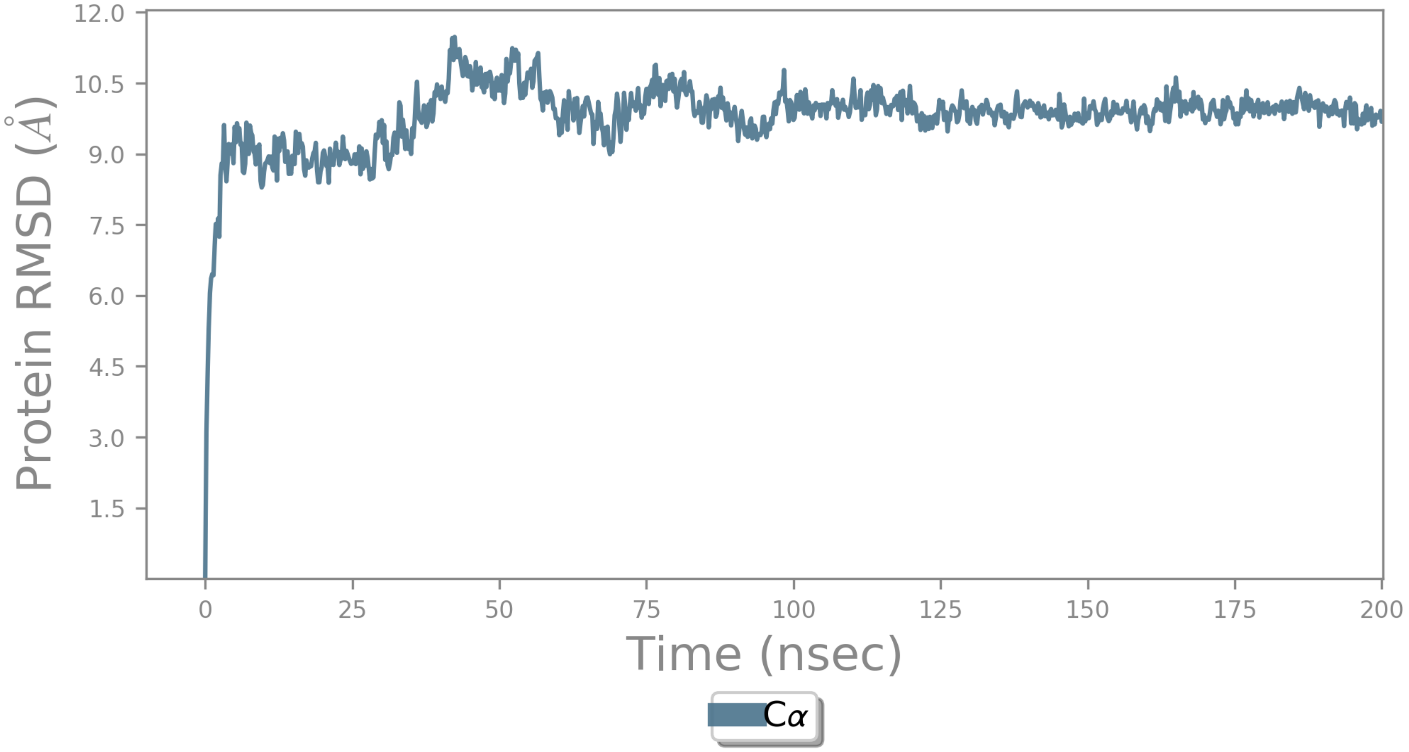

The Root Mean Square Deviation (RMSD) is used to measure the average change in displacement of a selection of atoms for a particular frame with respect to a reference frame. It is calculated for all frames in the trajectory. The RMSD for frame *x* is:

$$RMSD_x = \sqrt{\frac{1}{N}\sum_{i=1}^{N}(r'_i(t_x)) - r_i(t_{ref}))^2}$$

where N is the number of atoms in the atom selection; t_{ref} is the reference time, (typically the first frame is used as the reference and it is regarded as time t=0); and r' is the position of the selected atoms in frame x after superimposing on the reference frame, where frame x is recorded at time t_x. The procedure is repeated for every frame in the simulation trajectory.

Protein RMSD: The above plot shows the RMSD evolution of a protein (left *Y*-axis). All protein frames are first aligned on the reference frame backbone, and then the RMSD is calculated based on the atom selection. Monitoring the RMSD of the protein can give insights into its structural conformation throughout the simulation. RMSD analysis can indicate if the simulation has equilibrated — its fluctuations towards the end of the simulation are around some thermal average structure. Changes of the order of 1-3 Å are perfectly acceptable for small, globular proteins. Changes much larger than that, however, indicate that the protein is undergoing a large conformational change during the simulation. It is also important that your simulation converges — the RMSD values stabilize around a fixed value. If the RMSD of the protein is still increasing or decreasing on average at the end of the simulation, then your system has not equilibrated, and your simulation may not be long enough for rigorous analysis.

Protein RMSF

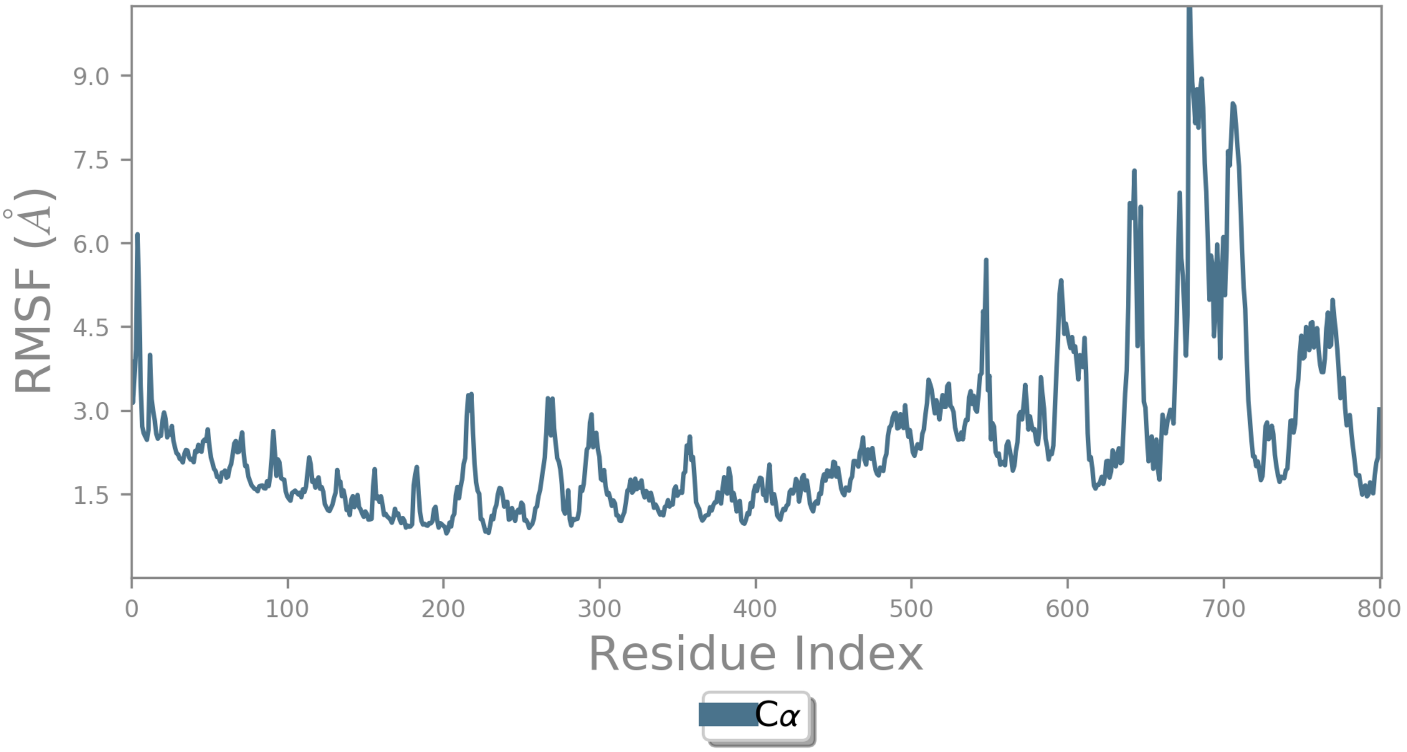

The Root Mean Square Fluctuation (RMSF) is useful for characterizing local changes along the protein chain. The RMSF for residue *i* is:

$$RMSF_i = \sqrt{\frac{1}{T}\sum_{t=1}^{T} < (r_i'(t)) - r_i(t_{ref}))^2 >}$$

where T is the trajectory time over which the RMSF is calculated, t_{ref} is the reference time, r_i is the position of residue i; r' is the position of atoms in residue i after superposition on the reference, and the angle brackets indicate that the average of the square distance is taken over the selection of atoms in the residue.

On this plot, peaks indicate areas of the protein that fluctuate the most during the simulation. Typically you will observe that the tails (*N*- and *C*-terminal) fluctuate more than any other part of the protein. Secondary structure elements like alpha helices and beta strands are usually more rigid than the unstructured part of the protein, and thus fluctuate less than the loop regions.

Protein Secondary Structure

% Helix	% Strand	% Total SSE
13.66	13.77	27.44

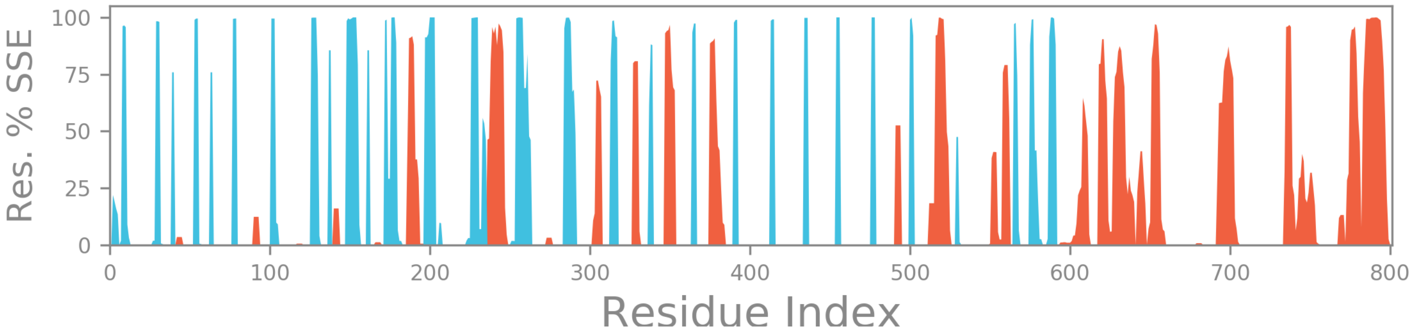

Protein secondary structure elements (SSE) like alpha-helices and beta-strands are monitored throughout the simulation. The plot above reports SSE distribution by residue index throughout the protein structure. The plot below summarizes the SSE composition for each trajectory frame over the course of the simulation, and the plot at the bottom monitors each residue and its SSE assignment over time.

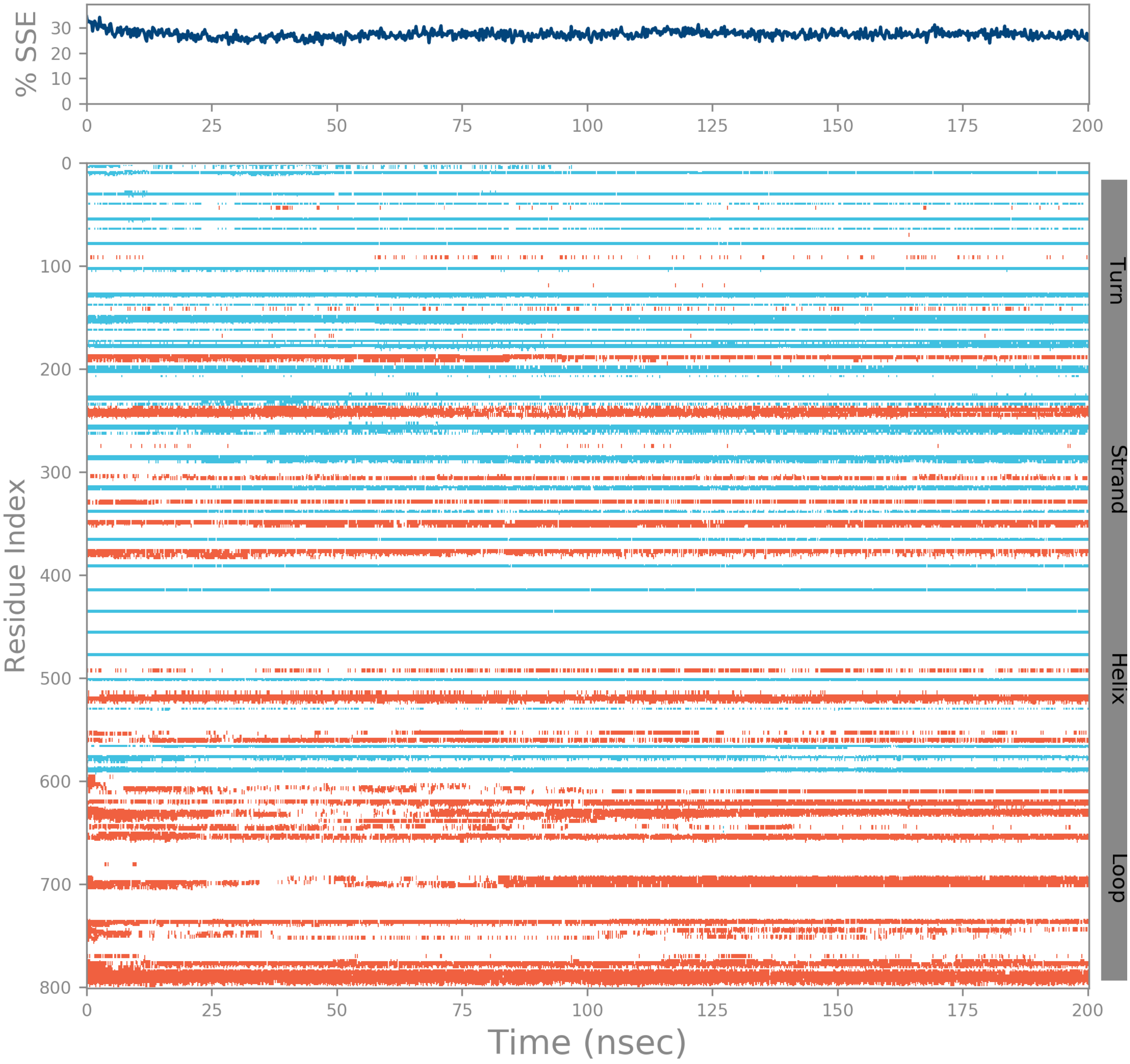
% SSE
30
20
10
0
0
25
50
75
100
125
150
175
200
Residue Index
0
100
200
300
400
500
600
700
800
Time (nsec)
Turn
Strand
Helix
Loop
