# Supplementary material for: In Silico Design and Evaluation of a Multi-Epitope Vaccine Candidate Against Staphylococcus haemolyticus
Source: Microorganisms. 2026 Sep 24;14(10):2152. doi: 10.3390/microorganisms14102152 (PMC13707924; doi:10.3390/microorganisms14102152)
Supplement: Supplementary file 1 [file microorganisms-14-02152-s001.zip › Supplementary Material S4/Vaccine 3D_Model/Galaxyrefine results.pdf]

Model	GDT-HA	RMSD	MolProbity	Clash score	Poor rotamers	Rama favored
Initial	1.0000	0.000	2.322	1.8	8.2	83.3
MODEL 1	0.9055	0.594	1.119	3.3	0.5	99.2
MODEL 2	0.9262	0.520	1.045	2.5	1.0	99.6
MODEL 3	0.9262	0.516	1.144	3.5	0.0	99.2
MODEL 4	0.9075	0.554	1.167	3.8	0.0	98.8
MODEL 5	0.9193	0.517	1.037	2.5	0.0	99.6
