# Supplementary material for: In Silico Design and Evaluation of a Multi-Epitope Vaccine Candidate Against Staphylococcus haemolyticus
Source: Microorganisms. 2026 Sep 24;14(10):2152. doi: 10.3390/microorganisms14102152 (PMC13707924; doi:10.3390/microorganisms14102152)
Supplement: Supplementary file 1 [file microorganisms-14-02152-s001.zip › Supplementary Material S4/Vaccine construct_FASTA.pdf]

GIINTLQKYYCRVRGGRCAVLSCLPKEEQIGKCSTRGRKCCRRKKEAAAKDQQAGASL
YAAYVAIDGLNLYAAYTADDFVYAWAAYSQTSLHHELAAYRQKSDLNLFAAYSVFTV
LIAIGPGPGQNGLVKQTSKSNSQQGPGPGVNKYKNSKALNHRLTGPGPGVEYKGDVAHI
FFHPLGPGPGTVDYGAAHAAKYGHEKKRETGKKRKKKNTSSNEINPKKNTQNSDNSEA
AAKAKFVAAWTLKAAAHHHHHH
